# *Helicobacter pylori* promotes gastric cancer through CagA-mediated mitochondrial cholesterol accumulation by targeting CYP11A1 redistribution

**DOI:** 10.7150/ijbs.96425

**Published:** 2024-07-15

**Authors:** Zhijun Zhang, Hongxin Huang, Zetian Chen, Mengpei Yan, Chen Lu, Zekuan Xu, Zheng Li

**Affiliations:** 1 Department of General Surgery, The First Affiliated Hospital of Nanjing Medical University, Nanjing, Jiangsu 210029, P. R. China.; 2 Gastric Cancer Center, The First Affiliated Hospital of Nanjing Medical University, Nanjing, Jiangsu 210029, P. R. China.; 3 Institute for Gastric Cancer Research, Nanjing Medical University, Nanjing, Jiangsu 211166, P. R. China.; 4 Jiangsu Key Lab of Cancer Biomarkers, Prevention and Treatment, Collaborative Innovation Center for Personalized Cancer Medicine, Nanjing Medical University, Nanjing, Jiangsu 211166, P. R. China.

**Keywords:** *Helicobacter pylori*, gastric cancer, cholesterol, translocation

## Abstract

Cholesterol and *Helicobacter pylori* (*H. pylori*) are both risk factors for gastric cancer (GC). However, the relationship between cholesterol and *H. pylori* and their function in the progression of GC are controversial. In this study, we addressed that *H. pylori* could induce mitochondrial cholesterol accumulation and promote GC proliferation and protect GC cells against apoptosis via cholesterol. Metabolomic and transcriptomic sequencing were used to identify CYP11A1 responsible for *H. pylori*-induced cholesterol accumulation. *In vitro* and *in vivo* function experiments revealed that cholesterol could promote the proliferation of GC and inhibit apoptosis. Mechanically, the interaction of Cytotoxin-associated gene A (CagA) and CYP11A1 redistributed mitochondrial CYP11A1 outside the mitochondria and subsequently caused mitochondrial cholesterol accumulation. The CYP11A1-knockdown upregulated cholesterol accumulation and reproduced the effect of cholesterol on GC in a cholesterol-dependent manner. Moreover, CYP11A1-knockdown or *H. pylori* infection inhibited mitophagy and maintained the mitochondria homeostasis. *H. pylori* could contribute to the progression of GC through the CagA/CYP11A1-mitoCHO axis. This study demonstrates that *H. pylori* can contribute to the progression of GC via cholesterol, and eradicating *H. pylori* is still prognostically beneficial to GC patients.

## Introduction

Gastric cancer (GC) is the fifth most prevalent malignancy worldwide and holds the fourth-highest global mortality rate^1^, ^2^. Notably, GC exhibits a higher prevalence in less developed countries than more developed ones, with about half of the world's GC cases occurring in East Asia, especially in China^3^. In addition, the insidious symptoms of GC in its early stages often result in most cases being initially diagnosed at advanced stages^4^. Therefore, most patients diagnosed with GC for the first time have a poor prognosis as they miss the opportunity for the most effective treatment. Despite the constant emergence of new treatment regimens, the prognosis for GC remains unsatisfactory, with a 5-year combined survival rate of less than 30%^5^. Therefore, investigators must explore additional mechanisms underlying the malignant progression of GC, as this will lay the foundation for developing more effective treatment regimens for GC.

*H. pylori* is a gram-negative, microaerophilic flagellated bacterium classified as a Class I carcinogen of GC by the World Health Organization (WHO)^6^. Investigators have reported that GC develops in approximately 3% of *H. pylori*-infected patients^7^. *H. pylori* can survive in the harsh gastric acid environment and cause epithelial lesions through various mechanisms^8^-^11^. For instance, to resist the robust acid environment, *H. pylori* can utilize its urease to degrade urea into ammonia, effectively neutralizing gastric acid^12^. Additionally, *H. pylori* can damage the epithelium and impair acid production through its virulence factors, cytotoxin-associated gene A (CagA), and vacuolating cytotoxin A (VacA)^12^, ^13^. Especially, *CagA* has the unique property of translocation into epithelial cells by the type IV secretion system (T4SS) of *H. pylori* and can interact with multiple intracellular molecules to exert its oncogenic effect^14^-^16^.

Beyond its role in the oncogenesis of GC, *H. pylori* is associated with the progression of GC^14^-^20^. For instance*, H. pylori* can promote the hyperproliferation of gastric epithelial AGS cells by activating NF-κB and AP-1^20^. Besides, research has indicated that *H. pylori* could contribute to the invasion and metastasis of GC by heparinase and thus endanger the prognosis of GC^19^. Furthermore, several clinical trials have demonstrated a favorable synergistic effect of anti-*H. pylori* therapy and GC treatment^17^, ^21^-^24^. However, the beneficial impact of eradicating *H. pylori* infection on the prognosis of GC remains contentious due to several clinical studies indicating better survival rates in the *H. pylori*-positive subgroup of GC patients^25^-^27^. Therefore, it is essential to delve into the underlying mechanism of *H. pylori*-induced progression of GC in a basic experiment.

The progression of GC involves a multifaceted process in which the interactions between GC cells and the surrounding tumor microenvironment (TME) play a crucial role^28^-^32^. The TME encompasses a spectrum of components, including the extracellular matrix (ECM), fibroblasts, immune cells, vasculature, mesothelial cells, and their metabolites^33^.

Tumor cells produce a variety of metabolites due to their abnormally high energy and anabolic metabolism, thereby remodeling the tumor microenvironment to make it more conducive to tumor cell growth^34^. Cholesterol is one of those abnormal metabolites in the TME, closely associated with GC^35^, ^36^. Specifically, there exists a notable correlation between cholesterol levels and GC, where lower high-density lipoprotein (HDL) cholesterol and elevated low-density lipoprotein (LDL) cholesterol are positively linked to the risk of GC^35^.

Remarkably, *H. pylori* infection is linked to elevated levels of total cholesterol (TC) and LDL cholesterol, along with reduced levels of HDL^37^-^40^, which hints that *H. pylori* may indirectly impact GC progression by causing cholesterol accumulation. However, as of now, no studies have elucidated the precise mechanism through which *H. pylori* contributes to the advancement of GC via cholesterol modulation.

Our study proved that *H. pylori* caused CYP11A1 translocation from the mitochondria to the cytoplasm via *CagA*-CYP11A1 interaction. Furthermore, such interaction can rearrange the distribution of CYP11A1, leading to CYP11A1 ectopic accumulation. Simultaneously, cholesterol would accumulate in mitochondria due to the decrease of mitochondrial CYP11A1 after translocation. Finally, this accumulation could enhance mitophagy, promoting GC progression by increasing cellular resistance.

## Results

### *H. pylori* infection contributed to cholesterol accumulation

Growing evidence suggests that *H. pylori* and metabolism are two major factors influencing GC progression^35^, ^41^-^43^. However, there is no clear evidence regarding the metabolites by which *H. pylori* can contribute to the progression of GC.

**Figure 1:**
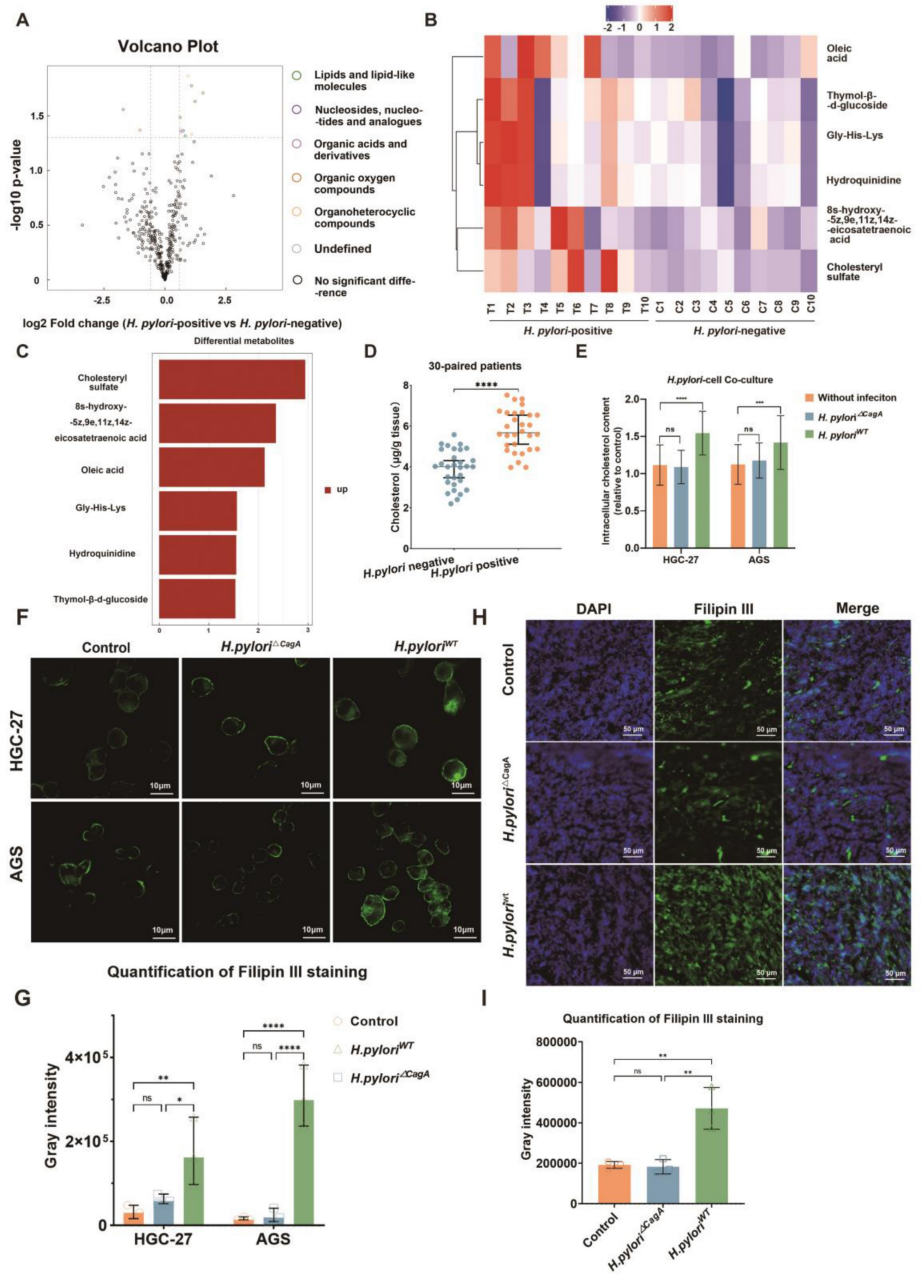
***H. pylori* contributed to cholesterol accumulation in a CagA-dependent manner. A** Volcano showed the differential metabolites (FC > 1.5 or FC<0.67, *p*< 0.05) from metabolomics sequencing of 10 pairs of *H. pylori*-positive and -negative GC tissues. **B-C** Heatmap (B) and histogram (C) were used to show the most significantly differential metabolites in *H. pylori*-positive GC tissues compared to those in *H. pylori*-negative GC tissues. **D** Cholesterol content examined by Elisa at the tissue level in 30 pairs of surgical specimens of human GC with or without *H. pylori* infection. **E-G** Intracellular cholesterol content was determined by Elisa (E) and Filipin III staining (F-G) in GC cells alone or co-cultured with different *H. pylori* strains. **H-I** Representative image of Filipin III staining of the frozen section was used to show the cholesterol content in subcutaneous tumors constructed from HGC-27 cells alone or co-cultured with different *H. pylori* strains. Data and error bars were shown as mean ± SD of triplicate independent replicate experiments. For the assessment of data passing independence, normality, and homogeneity of variance, the Student's t-test was employed to compare the differences between the two sets of data. Additionally, pairwise comparisons were conducted using one-way analysis of variance. Nonparametric tests were utilized in cases where the aforementioned conditions were not met. Significant flags and *p*-values are intricately linked in the following manner: (*p < 0.05; **p < 0.01; ***p < 0.001; ****p < 0.001).

Hence, we performed non-targeted metabolomic sequencing in 10 pairs of *H. pylori*-negative and positive human GC tissues to screen differentially distributed metabolites under different *H. pylori* infection states (**Figure 1A**) (Fold Change(FC) > 1.5 or < 0.67, *p* < 0.05). Among dysregulated metabolites, lipids and the cholesterol derivative cholesteryl sulfate were the most pronounced in *H. pylori*-positive GC tissue (**Figure 1B and 1C**). Notably, these two groups of patients had comparable serum cholesterol levels between *H. pylori*-negative and -positive subgroup and no patient had a history of dyslipidemia or had taken medication related to lipid regulation (**S1A, B**). The status of *H. pylori* was determined by the results of 13C breath test in medical record and Fluorescence *in situ* hybridization (FISH) staining with *H. pylori* probes (**S1C**).

To verify the clinical significance of *H. pylori* infection in GC, we analyzed the relationship between clinicopathologic data and *H. pylori* infection status in 433 GC patients with known *H. pylori* infection status (**Supplementary Table 1**). *H. pylori*-positive patients tended to have higher pre- and postoperative LDL cholesterol and total cholesterol. Collectively, these finds support that* H. pylori* infection could cause cholesterol accumulation in GC.

In order to eliminate the confounding factors of dyslipidemia, we examined the cholesterol content of GC tissue by ELISA in 30 pairs of GC patients with evenly distributed blood lipid levels in *H. pylori-*negative and -positive subgroups (**S1D, E**). The results showed that *H. pylori* infection could upregulate cholesterol content in GC tissues (**Figure 1D**).

Next, a co-culture model of human GC cell lines HGC-27 or AGS and *H. pylori* (100:1) was established (**Figure S1F**), and the changes of intracellular cholesterol before and after infection with *H. pylori* were measured by ELISA, which demonstrated that only CagA-positive *H. pylori* could cause significant upregulation of intracellular cholesterol in GC cells (**Figure 1E**). In addition, Filipin III staining of intracellular cholesterol demonstrated a similar tendency (**Figure 1F-G**). Finally, subcutaneous GC tumor models were constructed using AGS cells alone or pretreated with *H. pylori* strains with or without CagA deficiency (*H. pylori*^△CagA^*; H. pylori*^WT^). Then frozen sections were made from these subcutaneous tumors and subsequently stained for Filipin III. We observed a significant increase in cholesterol content in the *H. pylori*^WT^ subgroup (**Figure 1H-I**).

### Cholesterol promoted GC proliferation and inhibited apoptosis in GC cells

**Figure 2:**
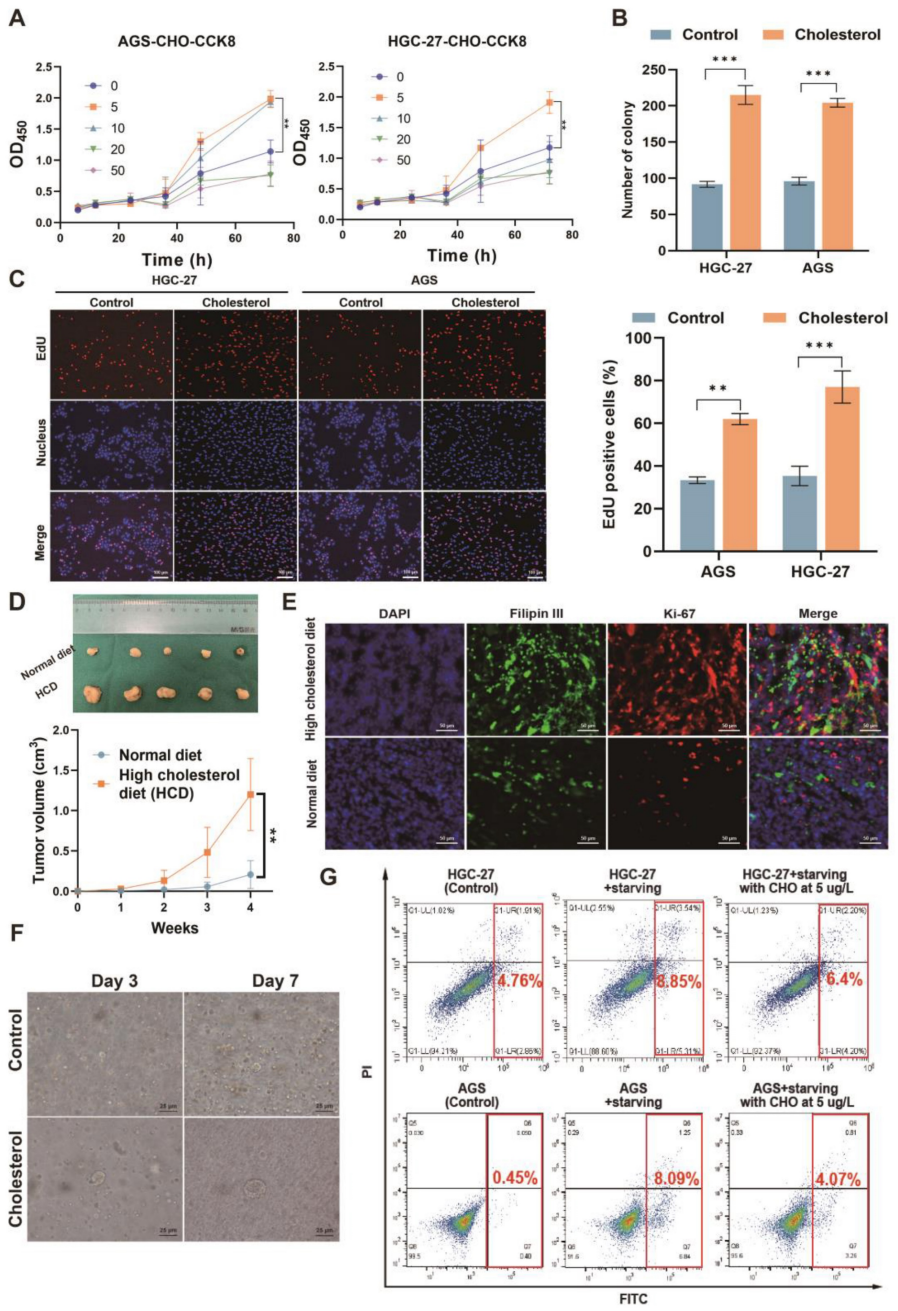
** Cholesterol promoted the proliferation of GC and protected GC cells against serum-induced apoptosis. A-C** The effect of cholesterol on proliferation was investigated by CCK8 (A), colony formation assay (B), and EdU assay (C). **D** The subcutaneous tumor-bearing mice chowed with high cholesterol diet (HCD) or regular diet (ND) were used to investigate the effect of cholesterol level on the proliferation of GC *in vivo*. **E** Representative images of Filipin III and Ki67 co-staining for subcutaneous tumor constructed in mice chowed with ND and HCD. **F** Microscopic bright-field images of GC organoid constructed from surgical specimens of patients with GC in the presence or absence of cholesterol (5 **μ**g/L). **G** The flow cytometry was used to investigate the influence of cholesterol on starvation-induced apoptosis. Data and error bars were shown as mean ± SD of triplicate independent replicate experiments. For the assessment of data passing independence, normality, and homogeneity of variance, the Student's t-test was employed to compare the differences between the two sets of data. Nonparametric tests were utilized in cases where the aforementioned conditions were not met. Significant flags and *p*-values are intricately linked in the following manner: (*p < 0.05; **p < 0.01; ***p < 0.001; ****p < 0.001).

Next, we investigated the impact of cholesterol on GC cells. Utilizing live-cell imaging, we observed that the cells were capable of taking up exogenously-added, fluorescently-labeled cholesterol (**Figure S1G**). This suggested that the extracellular addition of cholesterol could potentially influence the behavior and properties of the GC cells. In order to preliminarily determine the effect of cholesterol on cell proliferation *in vitro*, we investigated the effect of cholesterol on GC cell proliferation at different gradient concentrations (**Figure 2A**). The results showed that a cholesterol concentration of 5 ug/L had the strongest promoting effect on GC cell proliferation. Then we investigated the effect of *H. pylori* on the proliferation of GC cells via cholesterol using CCK8 (Cell Counting Kit 8) and EdU (5-Ethynyl-2'-deoxyuridine) experiments. The results showed that CagA-positive *H. pylori* (*H. pylori*^WT^) infection could promote the proliferation of GC cells (**Figure S2A-F**). However, this pro-proliferative effect could be eliminated after cholesterol removal by statins, which indicated that *H. pylori*^WT^ promoted the proliferation of GC cells, at least through cholesterol. Next, colony formation, and EdU experiments were performed in AGS and HGC-27 cells with or without cholesterol stimuli (5 μg/L)(**Figure 2B-C and S2G**). We observed that cholesterol could significantly promote proliferation in GC cells.

*In vivo*, subcutaneous tumor-bearing mice were randomized into the high-cholesterol diet (HCD) group and the normal diet (ND) group (n = 5/group). Our findings indicated that subcutaneous tumors exhibited accelerated growth in the HCD group, as evidenced by increased tumor volume (**Figure 2D**) and a higher percentage of Ki-67-positive cells(**Figure S2H**). Furthermore, we observed heightened proliferation in cholesterol-rich subcutaneous tumor tissues, as evidenced by the co-staining of Ki-67 and Filipin III, to assess the association between cholesterol content and tumor proliferation (**Figure 2E and Figure S2I**). Human GC tissues positive for *H. pylori* exhibited a higher proliferation rate than negative tissues (**Figure S2J**). Furthermore, the organoid models derived from surgical specimens of GC patients displayed enhanced growth upon adding cholesterol (**Figure 2F and Figure S2K**).

Finally, to mimic the harsh environment within which the GC is located, we treated GC cells with serum-free medium supplemented with or without cholesterol (5 ug/L) to induce apoptosis. We observed that cholesterol could increase resistance to apoptosis induced by the serum-free medium in GC cell lines (**Figure 2G and S2L-M**). In all, cholesterol was corroborated to promote the proliferation of GC and protect GC cells against apoptosis and *H. pylori* could promote GC via cholesterol.

### Identification of CYP11A1 responsible for mediating *H. pylori*-induced cholesterol accumulation in GC

**Figure 3:**
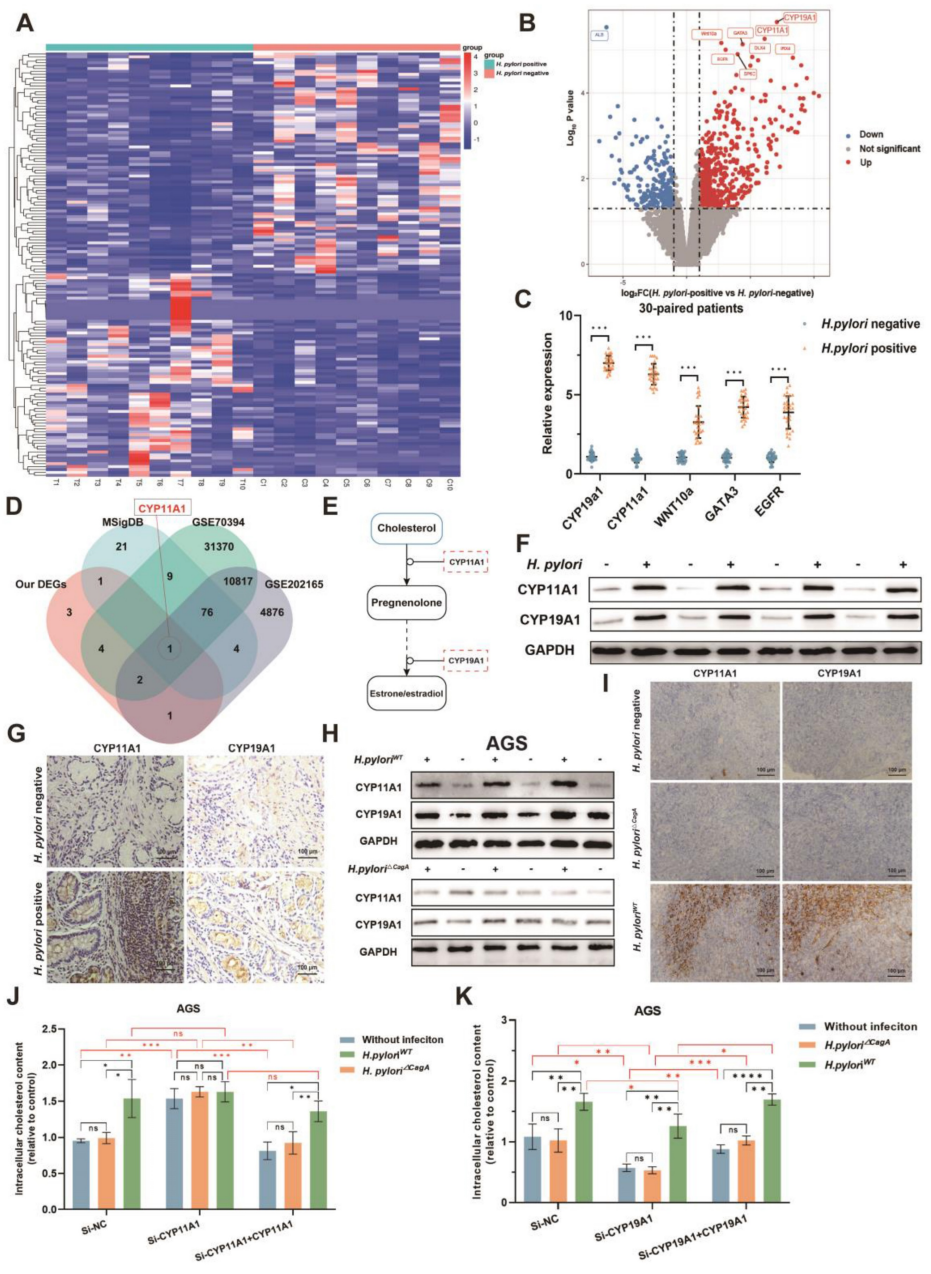
** CYP11A1 and CYP19A1 may mediate the *H. pylori*-induced cholesterol accumulation. A** Clustered heatmap showed the differentially expressed genes between *H. pylori*-positive and -negative GC tissues. **B** The volcano plots showed the expression variations of mRNAs in 10 *H. pylori*-positive GC tissues compared to matched 10 *H. pylori*-negative GC tissues. **C** PCR validated the top five most significantly upregulated genes in 30 pairs of human GC tissues. **D** Intersections were taken for the set of our differentially expressed genes and cholesterol-related gene sets (The Molecular Signatures Database, MSigDB), as well as for the differentially expressed genes between GC cells infected with *H. pylori* and those not infected from the GEO database. **E** KEGG pathway analysis of CYP11A1. **F-G** The expression CYP19A1 and CYP11A1 under different *H. pylori* infection status was examined by WB (F) and IHC (G) at the tissue level. **H** The expression of CYP11A1 and CYP19A1 were examined by WB in AGS cells alone and those infected by different *H. pylori* strains. **I** Subcutaneous tumors were constructed in nude mice (n=5 /group) from HGC-27 cells alone or co-cultured with different *H. pylori* strains. Then those tumors were subject to immunohistochemical staining to detect the expression of CYP11A1 and CYP19A1 (I). **J-K** The relative cholesterol content was varied in AGS cells by manipulating CYP11A1 (J) or CYP19A1 (K) under different *H. pylori* strains infection. Data and error bars were shown as mean ± SD of triplicate independent replicate experiments. For the assessment of data passing independence, normality, and homogeneity of variance, the Student's t-test was employed to compare the differences between the two sets of data. A mixed-design analysis of variance was used for pairwise comparisons. Nonparametric tests were utilized in cases where the aforementioned conditions were not met. Significant flags and p-values are intricately linked in the following manner: (*p < 0.05; **p < 0.01; ***p < 0.001; ****p < 0.001).

To determine the crucial genes in cholesterol accumulation induced by *H. pylori*, we performed transcriptomics sequencing of corresponding 10 pairs of *H. pylori*-negative and -positive GC surgical specimens from the same patients cohort as metabolomics sequencing (**Figure 3A-B**). A total of 12 differentially expressed genes (DEGs) were identified (|log_2_ FC|>1, *p*<0.05, FDR<0.05). Subsequently, we detected gene expression in 30 pairs of *H. pylori*-negative and -positive GC tissues by PCR, verifying the top 5 significantly differentially expressed genes. The results showed that all these genes were upregulated upon *H. pylori* infection, corroborating the results obtained from the sequencing data (**Figure 3C**).

Next, we screened the DEGs (|FC| > 1.5 and *p* < 0.05), which distinguished GC cells infected with *H. pylori* from the uninfected group using the GEO2R tool from the GEO database (GSE70394 and GSE202165). After intersecting these dysregulated genes with the cholesterol-related gene sets obtained from the Molecular Signature Database (MSigDB) (https://www.gsea-msigdb.org/gsea/msigdb) and our tissue sequencing data, CYP11A1 in the final intersection, indicating that it is one of the pivotal genes that are most likely responsible for *H. pylori*-induced cholesterol accumulation (**Figure 3D**). We found CYP11A1 could encode cholesterol side-chain cleavage enzyme (a rate-limiting enzyme), which could decompose cholesterol into progesterone by the KEGG database (**Figure 3E**) (https://www.genome.jp/kegg/). Another differentially expressed gene, CYP19A1, is the most upregulated in our data and is located downstream of CYP11A1, which is responsible for transforming progesterone into estrogen. Considering the close relation between CYP11A1 and CYP19A1, both genes were skeptical to play a role in cholesterol regulation.

Next, we further verified the protein expression of CYP11A1 and CYP19A1 under the induction of *H. pylori* infection (**Figure 3F-I and Figure S3A-B**). In the human GC tissue level, both CYP11A1 and CYP19A1 were upregulated by *H. pylori* infeciton according to WB and immunochemistry (**Figure 3F-G and Figure S3A-B**). At the cell level, we found that *H. pylori* induced CYP11A1 and CYP19A1 in a CagA-dependent manner (**Figure 3H and Figure S3C**). We also observed similar results in subcutaneous tumors constructed from HGC-27 cells alone or those co-cultured with different *H. pylori* strains by immunochemistry (**Figure 3I and Figure S3D**). In addition, the existence of exogenous CagA protein was examined in cell infected by different *H. pylori* strains using WB (**Figure S3E**).

Finally, to explore the functions of these two targets in mediating *H. pylori*-induced cholesterol accumulation, we use siRNA and overexpression plasmid to manipulate the expression of CYP11A1 and CYP19A1 in AGS or HGC-27 cells. These cells were subsequently co-cultured with different *H. pylori* strains, followed by examining the cholesterol content by ELISA. The results revealed that only *H. pylori*^WT^ could upregulate intracellular cholesterol in the presence of CYP11A1, indicating that *H. pylori* upregulated cholesterol in a CagA-dependent manner. Additionally, CYP11A1 knockdown would disrupt *H. pylori*^WT^-induced cholesterol accumulation (**Figure 3J and S3F**). In contrast, the cholesterol upregulation by *H. pylori*^WT^ was not disrupted by varying CYP19A1 expression (**Figure 3K and S3G**).

To sum up, we identified CYP11A1 as a critical target mediating *H. pylori*-induced cholesterol accumulation in GC cells in a CagA-dependent manner.

### CagA broke the negative regulatory relationship between CYP11A1 and CYP19A1

**Figure 4:**
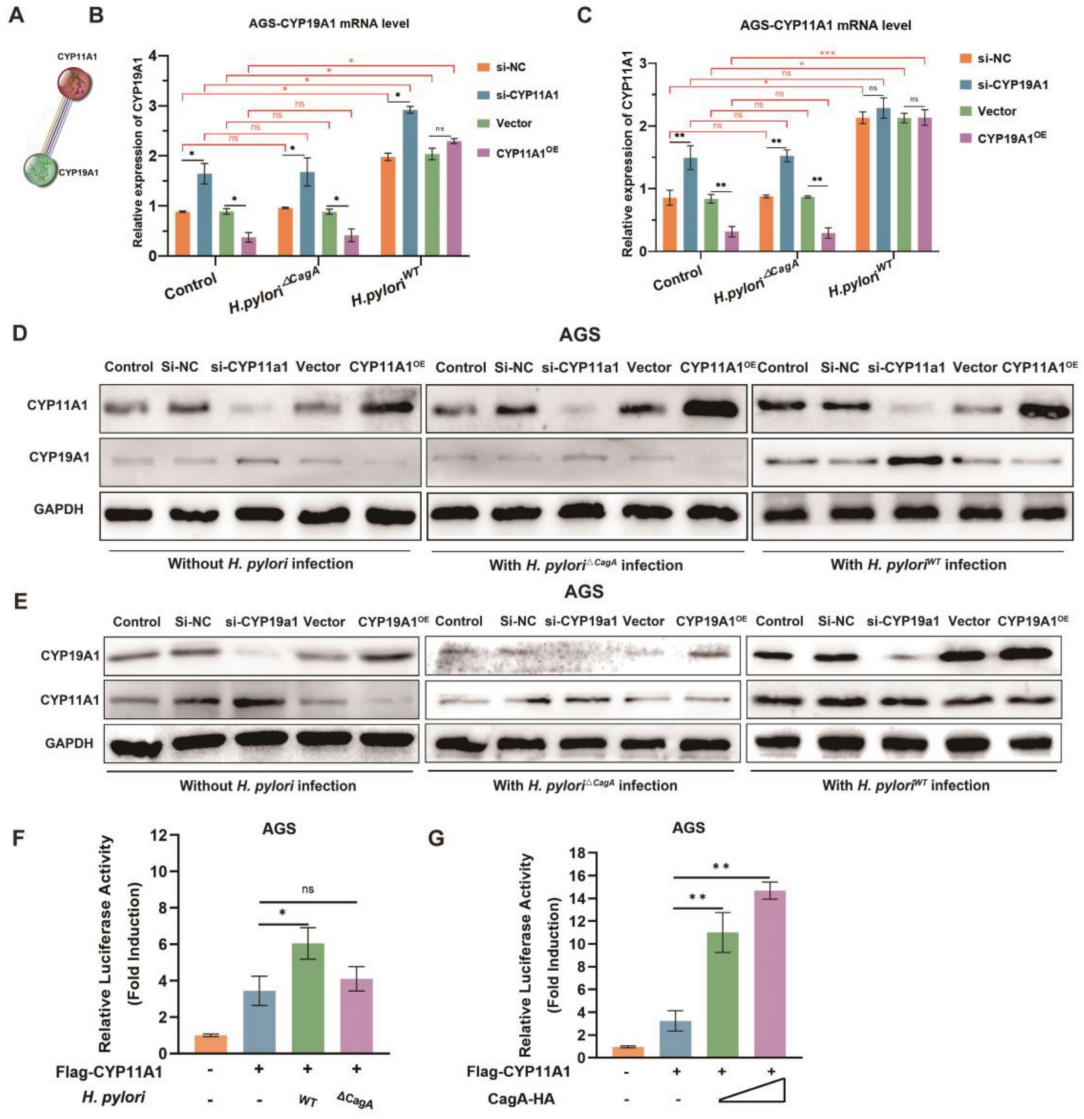
***H. pylori* disrupted the negative regulatory relationships between CYP11A1 and CYP19A1 in a CagA-dependent manner. A** The string database predicted a strong correlation between CYP11A1 and CYP19A1. **B** The effects of different CYP11A1 levels on CYP19A1 in AGS cells alone or pretreated by various *H. pylori* strains infection by PCR. **C** The influence of CYP19A1 on CYP11A1 in AGS cells alone or infected by different *H. pylori* strains by PCR. **D** The effects of CYP11A1 on CYP19A1 in AGS cells or those infected with different *H. pylori* strains by WB. **E** The effects of CYP19A1 on CYP11A1 in AGS cells or those infected with different *H. pylori* strains by WB.** F** Luciferase reporter assay was conducted in AGS cells infected by different *H. pylori* strains. **G** Luciferase reporter assay was performed in AGS cells transfected with indicated plasmids for 48 h. Data and error bars were shown as mean ± SD of triplicate independent replicate experiments. For the assessment of data passing independence, normality, and homogeneity of variance, the Student's t-test was employed to compare the differences between the two sets of data. A mixed-design analysis of variance or one-way analysis of variance was used for pairwise comparisons. Nonparametric tests were utilized in cases where the aforementioned conditions were not met. Significant flags and p-values are intricately linked in the following manner: (*p < 0.05; **p < 0.01; ***p < 0.001; ****p < 0.001).

Given the close regulatory relationship between upstream and downstream genes located in the same pathway, CYP11A1 and CYP19A1 were potential to directly or indirectly mediate *H. pylori*-induced cholesterol accumulation. The string database predicts a strong interaction between CYP11A1 and CYP19A1 (**Figure 4A**). Hence, we couldn't determine whether *H. pylori*^WT^ affected cholesterol directly through CYP11A1 or indirectly via the CYP19A1-CYP11A1 pathway. It is therefore necessary to explore the mutual regulation between CYP11A1 and CYP19A1 under different *H. pylori* infection to determine which one is the initial factor triggered by *H. pylori* infection.

AGS and HGC-27 cells with CYP11A1 knockdown or overexpressed were subjected to co-culturing with *H. pylori*^△CagA^ or *H. pylori*^WT^. Subsequently, the mutual interactions were examined at the mRNA level and protein level. In the control group or *H. pylori*^△CagA^ group, we identified the negative relationships between CYP11A1 and CYP19A1 by PCR (**Figure 4B-C and Figure S4A-B**)and WB (**Figure 4D-E and Figure S4C-D**). In contrast, *H. pylori*^WT^ infection partly disrupted the negative impact of CYP11A1 on CYP19A1: CYP11A1 knockdown could still upregulate CYP19A1; CYP11A1 by PCR overexpression could not downregulate CYP19A1(**Figure 4B and Figure S4A**) and WB (**Figure 4D and Figure S4C**).

In addition, *H. pylori*^WT^ infection totally disrupted the negative impact of CYP19A1 on CYP11A1: CYP11A1 was immune from the influence of CYP19A1 and could maintain a high expression level in GC cells infected with *H. pylori*^WT^(**Figure 4C**, **Figure 4E**, **Figure S4B** and **Figure S4D**). Besides, we found that CYP19A1 and CYP11A1 were upregulated in the *H. pylori*^WT^ subgroup compared to the other two groups (control or *H. pylori*^△CagA^), suggesting this up-regulation was CagA-dependent. These results can be explained by that *H. pylori*^WT^ could upregulate CYP11A1 directly and independently and CYP19A1 could not impact CYP11A1 in *H. pylori*^WT^ group. Furthermore, CYP11A1-overexpression could no longer decrease CYP19A1 once infected by *H. pylori^WT^*, while CYP11A1-knockdown could still up-regulate CYP19A1 in GC cell with *H. pylori^WT^* infection (Figure 4B, 4D, S4A and s4C), indicating *H. pylori^WT^* may exert an indirect impact on CYP19A1 expression via CYP11A1. Above all, we excluded the CYP19A1-CYP11A1 pathway in response to *H. pylori*^WT^ infection.

To verify the direct induction of CYP11A1 by *H. pylori*^WT^, we conducted luciferase assays and found that CYP11A1 was induced significantly when the GC cells were infected with *H. pylori*^WT^ or transfected with the CagA plasmid (**Figure 4F-G and S4E-F**).

Here, we excluded the indirect influence of CYP19A1 and finally identified CYP11A1 as a key target responsible for the direct induction of cholesterol accumulation by *H. pylori* in a CagA-dependent manner.

### CYP11A1 impacted the proliferation and apoptosis of GC by regulating cholesterol

**Figure 5:**
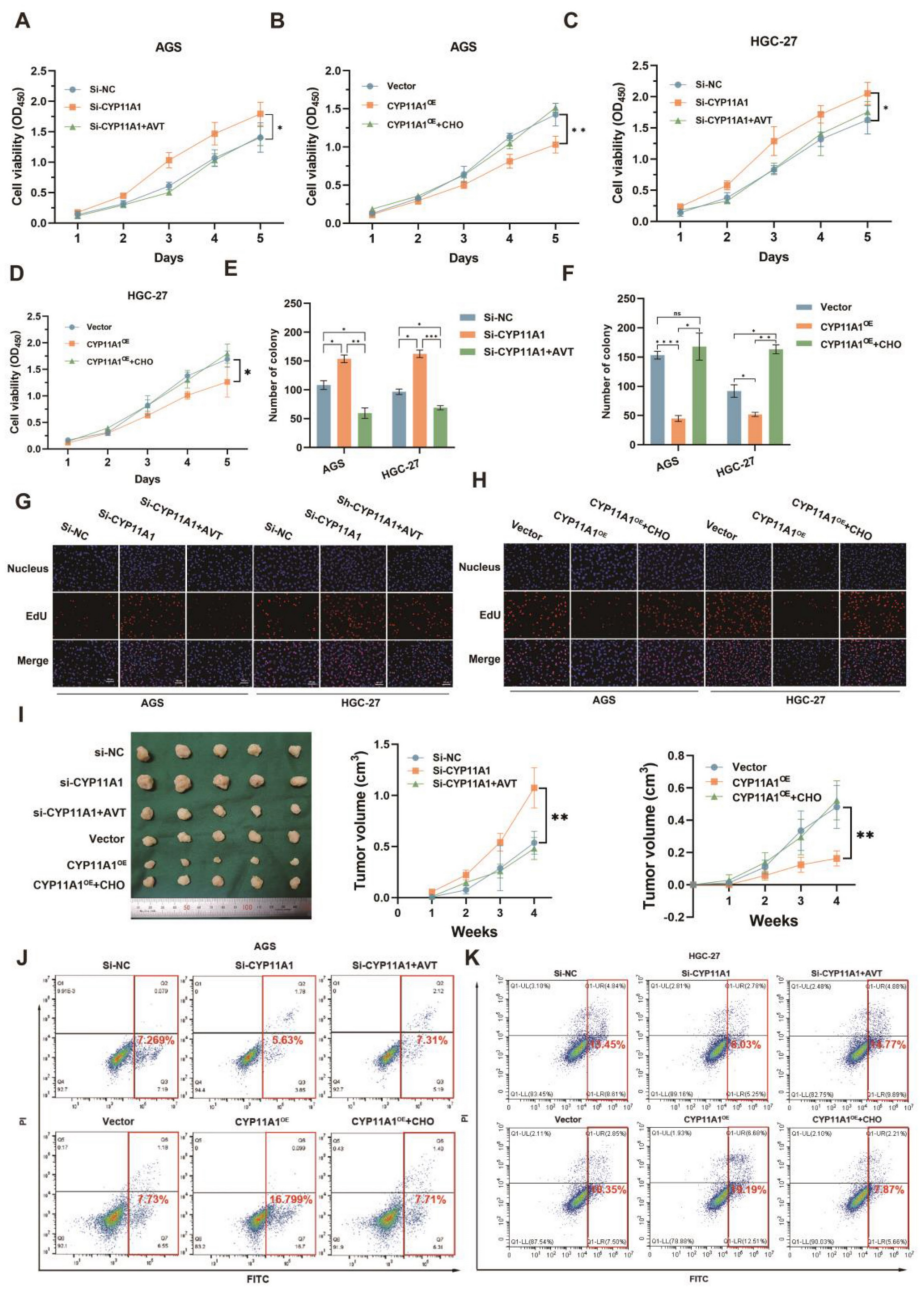
** CYP11A1 could impact the proliferation and apoptosis of GC cells by negatively regulating cholesterol content *in vivo* and *in vitro*.** AGS and HGC-27 cells were transfected with si-CYP11A1, CYP11A1, and corresponding controls. Then AVT was administered in cells with CYP11A1-knockdown to deplete mitochondrial cholesterol. Moreover, cholesterol was used to counteract mitochondrial cholesterol exhaustion by CYP11A1-overexpression in corresponding cells. **A-D** CCK8 assays were performed in AGS cells with CYP11A1-knockdown (A) and -overexpression (B) and in HGC-27 cells with CYP11A1-knockdown (C) or -overexpression (D). **E-F** Colony formation assays were performed in AGS and HGC-27 cells with CYP11A1-knockdown (E) or CYP11A1-overexpression (F). **G-H** EdU experiments were conducted in AGS and HGC-27 cells with CYP11A1-knockdown (G) and -overexpression (H). **I** Subcutaneous tumors were constructed from HGC-27 cells with CYP11A1-knockdown and -overexpression. As mentioned in the methods and materials, tumor-bear mice were treated with AVT and cholesterol for the knockdown and overexpression groups, respectively. **J-K** AGS cells (**J**) and HGC cells (**K**) were transfected with si-NC, si-CYP11A1 and then divided into three groups (si-NC group, si-CYP11A1 group, and si-CYP11A1+AVT group). Subsequently, they were treated with a serum-free medium for the indicated time, and then apoptosis rates were detected in each group by flow cytometry. Data and error bars were shown as mean ± SD of triplicate independent replicate experiments. For the assessment of data passing independence, normality, and homogeneity of variance, the Student's t-test was employed to compare the differences between the two sets of data. Additionally, pairwise comparisons were conducted using one-way analysis of variance. Nonparametric tests were utilized in cases where the aforementioned conditions were not met. Significant flags and p-values are intricately linked in the following manner: (*p < 0.05; **p < 0.01; ***p < 0.001; ****p < 0.001).

We have identified that *H. pylori*^WT^ could promote GC via upregulating cholesterol by CYP11A1. It was still unknown whether CYP11A1 could impact GC progression via cholesterol. To figure it out, we performed CCK8, colony formation and EdU assays in treated GC cells (**Figure 5A-H and Figure S5A-C**). CYP11A1-knockdown could promote the proliferation of GC cells, and cholesterol depletion by statin (atorvastatin, AVT) could counteract this promotion (**Figure 5A, C, E, G, and Figure S5A-B**). Conversely, CYP11A1 overexpression suppressed GC cell proliferation, and cholesterol could counteract this suppression (**Figure 5B, D, F, H and Figure S5A, C**).

*In vivo*, subcutaneous tumours were constructed from treated HGC-27 cells in nude mice (n=5/group) with AVT or high cholesterol diet (**Figure 5I left**). The diameters of the corresponding subcutaneous tumours were measured every week (**Figure 5I right**). We arrived at a similar conclusion that CYP11A1 suppressed GC via negatively regulating cholesterol.

The apoptosis assays revealed that CYP11A1-knockdown could suppress the serum-free medium-induced apoptosis in AGS and HGC-27 cells (**Figure 5J-K and Figure S5D-E**). AVT could counteract this suppression of apoptosis. On the contrary, CYP11A1-overexpression could strengthen the serum-free medium-induced apoptosis in both cells, and cholesterol supplementation could reverse this effect.

Collectively, we found that CYP11A1 and cholesterol are functionally opposite in affecting GC, and CYP11A1 exerted its function by negatively regulating cholesterol.

### CagA could directly bind to CYP11A1

*H. pylori* can induce upregulation of CYP11A1 at the mRNA and protein level, but the CYP11A1-encoded enzyme catabolizes cholesterol, contrary to the fact that *H. pylori* can induce cholesterol accumulation. In addition, we noted that *H. pylori* mediated both cholesterol and CYP11A1 upregulation in a CagA-dependent manner. Hence we next explored whether CagA could directly affect the function of CYP11A1 at the protein level.

**Figure 6:**
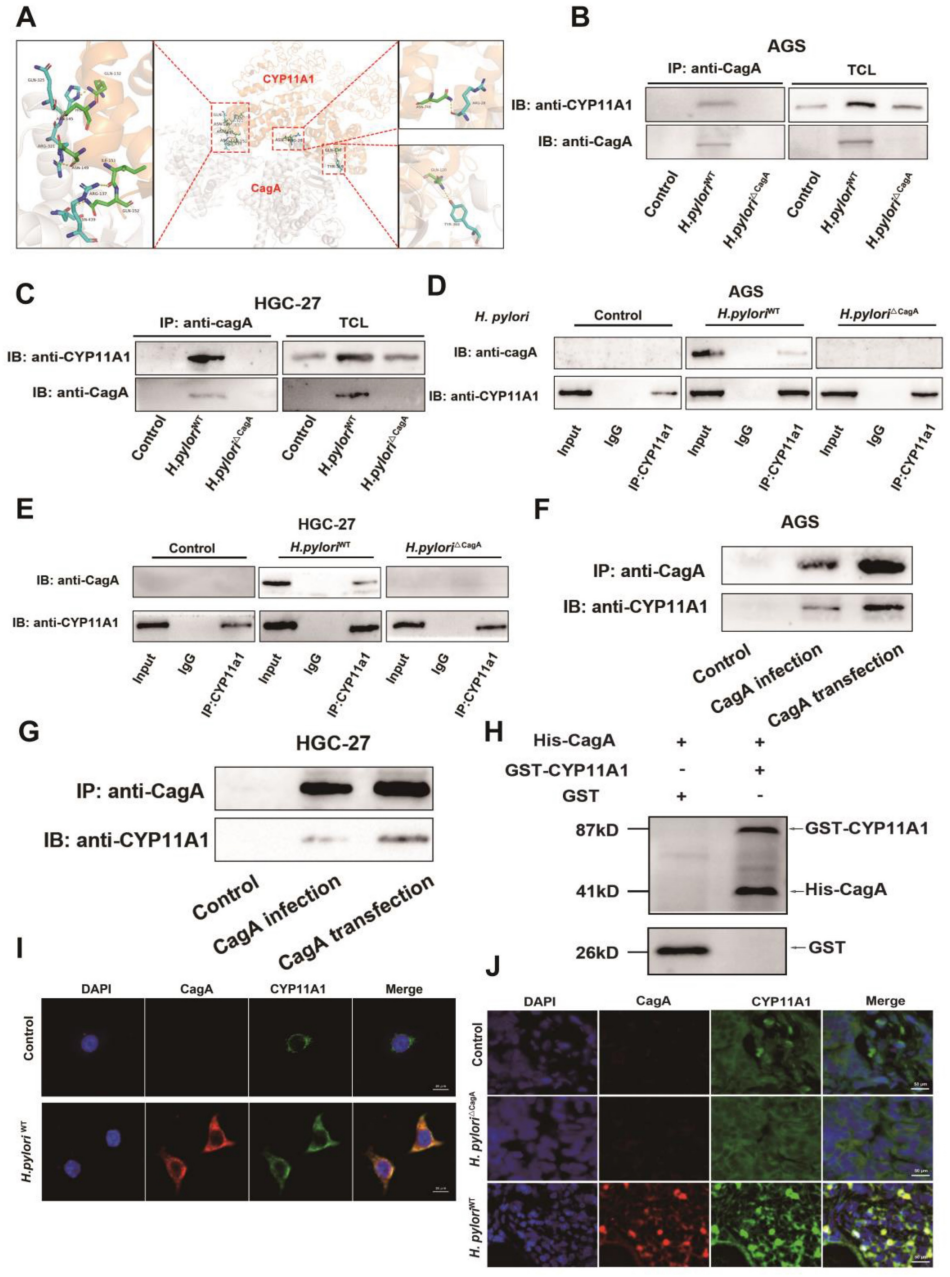
** The CagA protein could directly bind to CYP11A1 protein. A** The HDOCK protein docking database predicted the binding of CagA and CYP11A1. **B-E** Co-immunoprecipitation (Co-ip) assays were performed in different GC cells with or without *H. pylori* infection. AGS cells or HGC-27 cells were infected with different *H. pylori* strains alone or in combination with different *H. pylori* strains. Then the corresponding protein solutions were extracted and subjected to electrophoresis for the detection of the relevant molecules after immunoprecipitation with anti-CagA or anti-CYP11A1 magnetic beads. **F-G** Co-ip was performed in GC cells transfected with CagA plasmid or infected with CagA-positive *H. pylori* strains. **H** GST pull-down was performed to confirm the direct binding between CagA and CYP11A1. **I** Immunofluorescence staining (IF) of CagA and CYP11A1 was performed in GC cells and those infected by *H. pylori^WT^*. **J** Representative images of Immunofluorescence for CagA and CYP11A1 in subcutaneous tumors tissue constructed from HGC27 cells alone and those infected by different *H. pylori* strains.

First, we used the HDOCK online database (http://hdock.phys.hust.edu.cn/) to predict that CagA could bind directly to CYP11A through hydrogen bonds formed between several residues (**Figure 6A**). Then we further confirmed the interaction between CagA and CYP11A1 by performing bidirectional immunoprecipitation experiments in two types of GC cells (**Figure 6B-E**). Briefly, protein solutions from GC cells infected with *H. pylori* strains were subject to anti-CagA or anti-CYP11A1magnetic beads. Subsequently, the immunoprecipitated protein solutions underwent electrophoresis to detect the corresponding antibodies. The results revealed that CYP11A1 and CagA could interact with each other in GC cells with *H. pylori*^WT^ infection. Moreover, this interaction was demonstrated in cells transfected with CagA (**Figure 6F-G**). Finally, we found that CagA protein could directly bind to CYP11A1 by GST-pull down assay (**Figure 6H**).

To verify the colocalization between CagA and CYP11A1, we performed immunofluorescence staining in GC cells and mice subcutaneous tumour tissue (**Figure 6I-J**). The results showed that CagA and CYP11A1 overlapped and were localized in the cytoplasm (**Figure 6I-J**).

In this section, we confirmed that CagA could directly bind to CYP11A1 at the protein level.* H. pylori* induced mitochondrial cholesterol accumulation by CagA-mediated redistribution of CYP11A1 from mitochondria to the cytoplasm. The subcellular localization of the CagA protein is the cytoskeleton and cell membrane, and a minimal amount is located in the nucleus, while CYP11A1 is a typical mitochondrial inner membrane protein, which suggests the existence of redistribution of either protein^44^.

**Figure 7:**
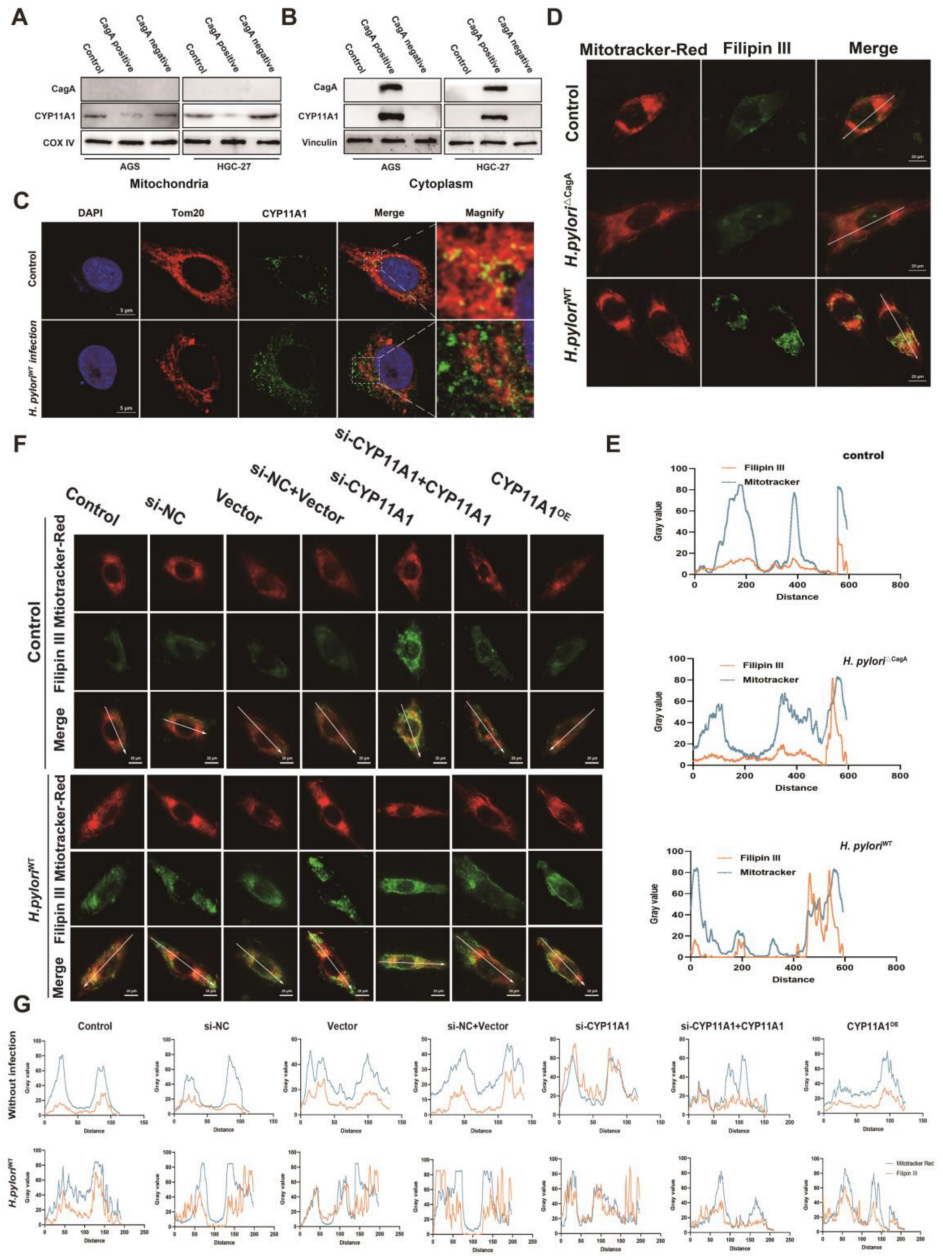
***Helicobacter pylori* induced the translocation of CYP11A1 protein out of mitochondria via CagA-CYP11A1 interaction and thus caused mitochondrial cholesterol accumulation. A-B** Mitochondrial proteins (A) and cytoplasmic proteins (B) isolated from GC cells infected or uninfected with different *H. pylori* strains were subject to WB experiments to detect CYP11A1 and CagA. **C** Laser confocal microscopy revealed CYP11A1 protein expression and subcellular localization in uninfected HGC-27 cells and those infected with *H. pylori*^WT^. **D-E** Mitotracker-Red and Filipin III co-staining represented mitochondrial cholesterol content in AGS cells either uninfected or infected with different *H. pylori* strains. **F** The mitochondrial cholesterol content represented by Mitotracker-red and Filipin III co-staining varied in represented AGS cells with either CYP11A1 manipulation or *H. pylori^WT^* infection. **G** Colocalization analysis of **F** was performed using Plot Profile of image J software.

To explore this, we investigated the distribution of these two proteins in the mitochondria and extramitochondrial cytoplasm. First, the mitochondrial isolation assays further revealed that the CYP11A1 translocated outside the mitochondria (**Figure 7A**) and accumulated in the cytoplasm (**Figure 7B**) under *H. pylori*^WT^ infection. Next, to investigate changes in CYP11A1 protein caused by *H. pylori*^WT^ infection, we used laser confocal microscopy to directly observe immunofluorescence staining of anti-CYP11A1 and anti-TOM20 antibodies (**Figure 7C**).

The findings indicated that *H. pylori*^WT^ infection not only led to an increase in the expression of CYP11A1 protein, but also resulted in a shift in its subcellular localization from the mitochondria to the cytoplasm. Although *H. pylori*^WT^ infection significantly upregulated the expression of CYP11A1, it was noteworthy that the majority of CYP11A1 would be redistributed outside the mitochondria and thus rendered inactive.

Then, to investigate the effect of CYP11A1 redistribution on cholesterol, we labelled mitochondrial cholesterol using mitochondrial probes and cholesterol probes. We found that mitochondrial cholesterol content was significantly upregulated in cells infected with *H. pylori*^WT^(**Figure 7D-E**). Furthermore, the mitochondrial cholesterol content was negatively associated with the expression of CYP11A1 in GC cells without *H. pylori*^WT^ infection (**Figure 7F-G**). Notably, *H. pylori*^WT^ could upregulate the content of mitochondrial cholesterol in all groups, except the si-CYP11A1 group. These findings indicated that *H. pylori*^WT^ infection primarily increased cellular mitochondrial cholesterol through CYP11A1.

Above all, we identified that CagA could cause the redistribution of CYP11A1 outside the mitochondria and subsequently mediated mitochondrial cholesterol accumulation. The ectopic CYP11A1 could not decompose cholesterol, which explained the cholesterol accumulation induced by *H. pylori*^WT^

### Mitochondrial cholesterol accumulation inhibited mitophagy

Mitochondria are crucial organelles to maintain cell survival. Hence, we next explored the effect of mitochondrial cholesterol accumulation caused by CYP11A1 redistribution on mitochondria. We manipulated the expression of CYP11A1 in GC cells, and then they were treated with a serum-free medium for the indicated time. CYP11A1 knockdown could cause cholesterol upregulation according to our results** (Figure 3J and S3F)**. Hence, we applied cholesterol or statin to counteract the cholesterol by CYP11A1 in the corresponding groups.

**Figure 8:**
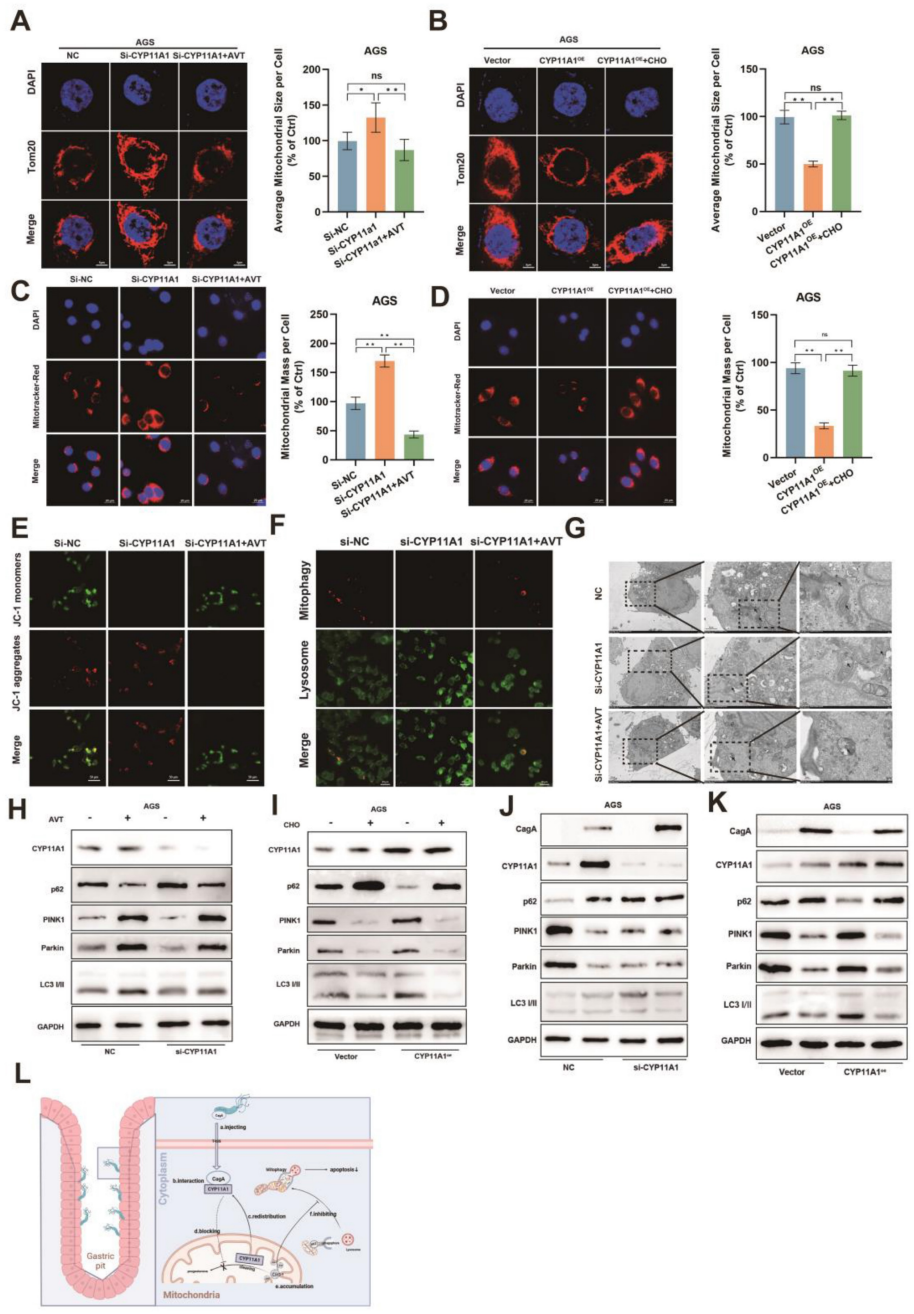
** CYP11A1 could regulate serum-free medium-induced cell mitophagy via changing cholesterol content.** AGS cells were transfected with si-CYP11A1 or CYP11A1 plasmid, followed by treatment with AVT or cholesterol to counteract the impact of CYP11A1 manipulation on cholesterol. Then these cells were treated with a serum-free medium for 12 h. Next, the following procedures were performed in those cells. **A-B** Immunofluorescence staining of anti-Tom20 was performed to represent the total mitochondrial size, and quantification was done in the indicated groups. **C-D** Mitotracker-Red staining (C) represented the functional mitochondrial in the indicated groups, and quantification was done in the corresponding groups. **E** JC-1 staining represented the mitochondrial potential of the knockdown AGS cells without or with statin (Atorvastatin, AVT) treatment. **F** Co-staining of mitophagy dye and lysosomal dye was used to detect the mitophagy. **G** Electron microscopy showed representative mitophagy. **H-I** Examination of PINK1, Parkin, p62 and LC3i/ii by WB in the knockdown group alone or with AVT stimuli (H) and in overexpression cells alone or with cholesterol stimuli (I). **J-K** Examination of PINK1, Parkin, p62 and LC3i/ii by WB in AGS cells with CYP11A1 knockdown (J) or overexpression (K) under the infection of *H. pylori*^WT^. **M** Mechanism diagram of this work. Generally, the interaction of CagA from *H. pylori* with CYP11A1 mediated the CYP11A1 redistribution outside the mitochondria and therefore caused mitochondrial cholesterol accumulation and subsequent mitophagy inhibition and tumor progression. Data and error bars were shown as mean ± SD of triplicate independent replicate experiments. For the assessment of data passing independence, normality, and homogeneity of variance, pairwise comparisons were conducted using one-way analysis of variance. Nonparametric tests were utilized in cases where the aforementioned conditions were not met. Significant flags and p-values are intricately linked in the following manner: (*p < 0.05; **p < 0.01; ***p < 0.001; ****p < 0.001).

Starvation could decrease the mitochondria size (**Figure S6A-B**). CYP11A1 knockdown increased cellular mitochondrial resistance to starvation treatment, but statins abrogated this resistance by cholesterol depletion (**Figure 8A and S6C-D**). On the contrary, CYP11A1 overexpression made cellular mitochondria more sensitive to starvation treatment with a significant decrease in mitochondria size, and cholesterol treatment could counteract this effect (**Figure 8B and S6E-F**).

The functional mitochondria, was shown by Mitotracker-Red staining. The functional mitochondrial significantly decreased in AGS cells with starvation treatment (**Figure S6G**). CYP11A1 knockdown protected AGS cells from starvation treatment with increased mitochondria mass (**Figure 8C**). In contrast, CYP11A1 overexpression was shown to sensitize AGS cells to starvation stimuli with a decrease in functional mitochondrial (**Figure 8D**). The treatment with AVT or cholesterol could counteract the corresponding impact of CYP11A1 on functional mitochondrial (**Figure 8C-D**). Moreover, the mitochondrial potential shown by JC-1 staining revealed a similar tendency to the change of mitochondrial size or mass regulated by CYP11A1 (**Figure 8E and Figure S6H**).

Mitophagy is essential for regulating mitochondrial homeostasis, so we further interrogated the effect of CYP11A1 on mitophagy in this starvation-induced Mitochondrial destabilization process. We found that CYP11A1 knockdown could inhibit starvation-induced mitophagy, which was abrogated by AVT (**Figure 8F**). On the other hand, CYP11A1 overexpression could increase the sensitivity of cells to starvation treatment and make them more prone to mitophagy (**Figure S6I**). Exogenous addition of cholesterol restored their resistance to starvation and made cells less prone to mitophagy (**Figure S6I**). Similar phenomena could be obtained by observing mitochondrial morphology and the formation of autophagosomes by electron microscopy (**Figure 8G and Figure S6J**) and examining mitophagy markers PINK1, Parkin, LC3 and p62 by WB (**Figure 8H-I and S6K-L**). The results showed that cholesterol inhibited the mitophagy induced by starvatioon and cholesterol depletion by statin would contribute to mitphagy (**Figure 8H-I and S6K-L**). We observed that CYP11A1 knockdown or overexpression could no longer impact mitophagy in cells treat with statin or cholesterol, indicating that CYP11A1 mainly impact mitophagy via cholesterol regulation. Given that *H. pylori*^WT^ infection could contribute to cholesterol accumulation via targeting CYP11A1 redistribution, we also examined these markers in GC cells with CYP11A knockdown or overexpression under *H. pylori*^WT^ infection or not (**Figure 8J-K and S6M-N**). We found that *H. pylori*^WT^ could inhibited mitophagy in GC cells in the presence of CYP11A1. When CYP11A1 was knockdown, *H. pylori*^WT^ could not remarkably inhibited mitophagy, which suggested that CYP11A1 mediated the mitophagy inhibition by *H. pylori*^WT^*.*

Collectively, these results confirmed that CYP11A1 could exacerbate mitophagy via exhausting cholesterol and adding cholesterol or AVT could counteract the effect of CYP11A1 manipulation on mitophagy. And we also demonstrated that *H. pylori*^WT^ could inhibit mitophagy by targeting CYP11A1.

## Discussion

Although *H. pylori* has been established as the Class I carcinogen of GC, its role in GC progression remains contentious^45^-^51^. We discovered that *H. pylori* could promote mitochondrial cholesterol accumulation by redistributing CYP11A1 outside the mitochondria via CagA-CYP11A1 interaction. Cholesterol was found to further the proliferation of GC, safeguard GC cells against apoptosis, and maintain mitochondrial homeostasis by inhibiting mitophagy.

*H. pylori* is closely associated with cholesterol metabolism^37^-^40^, ^52^, ^53^. A clinical study including 462 subjects from Korea reported that *H. pylori* infection could contribute to an increased LDL cholesterol level corresponding to infection severity^40^. Furthermore, an Italian study further demonstrated that *H. pylori* infection could heighten serum lipids to varying degrees, depending on CagA status^52^. Increased LDL levels, instrumental for cholesterol transport to peripheral tissues, were observed in *H. pylori*-positive GC patients, suggesting peripheral cholesterol accumulation 54. We also observed an elevated LDL level in *H. pylori*-positive GC patients compared to that in *H. pylori*-negative subgroup. Hence, we hypothesized that *H. pylori* could cause cholesterol accumulation in GC.

To authenticate our hypothesis, we first performed untargeted metabolomic sequencing of 10 sets of *H. pylori*-negative and -positive GC tissues and found that cholesterol accumulated substantially in *H. pylori*-positive GC tissues. Moreover, cholesterol ranked first among the upregulated differential metabolites. Furthermore, cholesterol accumulation induced by *H. pylori* was corroborated at various levels: human tissue, murine tissue, and GC cell level. This elevation was CagA-dependent, as previously stipulated^52^.

Indeed, several studies have reported that *H. pylori* could exert an effect on cholesterol metabolism via CagA in other cells^55^-^57^. For instance, in human liver cells, CagA was reported to initiate the ERK/MAPK pathway, potentially impacting the expression of genes associated with cholesterol synthesis and uptake. Notably, the activation of ERK/MAPK was shown to enhance the activity of SREBP (sterol regulatory element-binding proteins), a transcription factor that impacts the expression of pivotal genes involved in cholesterol synthesis and uptake, such as HMG-CoA reductase and LDL receptor^57^. Previous studies have demonstrated that infection with CagA-positive *H. pylori* or the presence of exosomal vesicles containing CagA, along with the introduction of CagA recombinant protein, could lead to significant cholesterol accumulation within infected macrophages and subsequent conversion into foam cells^55^. Extensive investigation has elucidated the underlying mechanism, revealing that CagA effectively promotes cholesterol buildup in macrophages by inhibiting the PPARγ-LXRα pathway, resulting in decreased expression of cholesterol efflux transporters (ABCA1/ABCG1/SR-BI)^55^. This evidence strongly supports our results that CagA-positive *H. pylori* infection significantly increase intracellular cholesterol in GC cells.

Next, to dissect the underlying mechanisms involved, transcriptome sequencing of these GC tissue pairs was performed and yielded relevant differentially expressed genes. We intersected them with the relevant differential gene sets of *H. pylori*-infected GC cells versus uninfected ones in the GEO database as well as the cholesterol metabolism disorder-related gene sets to obtain the key target, CYP11A1, which can encode cholesterol side chain cleaving enzymes that break down cholesterol or cholesterol sulfate to produce pregnenolone^58^. Notably, the initial attempt to explore the effect of CYP11A1 on intracellular cholesterol under different *H. pylori* infection status indicated that *H. pylori*^WT^ could positively regulate intracellular cholesterol in a CagA-dependent manner. However, CYP11A1 upregulated by *H. pylori*^WT^ was shown to downregulate cholesterol, contradicting cholesterol accumulation under *H. pylori*^WT^ infection.

CYP11A1 can encode cholesterol-side cleavage enzyme to decompose cholesterol or cholesterol sulfate into progesterone. Progesterone is the substrate for proteins encoded by another significantly differentially expressed gene, CYP19A1, to generate steroids^59^-^61^. Considering the potential interactions between these two genes, both targets should have been skeptical about being directly induced by *H. pylori* and then impacted the other.

Hence, we examined their interactions under different *H. pylori* infection status. Interestingly, we found that the regulation of CYP11A1 by CYP19A1 was utterly disrupted in the presence of *H. pylori*^WT^ infection, with CYP11A1 remaining at a high level without the impact of CYP19A1 in response to *H. pylori*^WT^ infection. In contrast, the negative regulation of CYP19A1 by CYP11A1 was only partially disrupted. These results suggest that *H. pylori*^WT^ could directly affect CYP11A1 to modulate cholesterol in a CagA-dependent manner, which corresponds precisely to the previous cholesterol accumulation evident in infected GC by *H. pylori*^WT^.

Furthermore, *H. pylori*^WT^ infection* and* CagA were found to promote CYP11A1 at the transcriptional level. However, this transcription upregulation of CYP11A1 by *H. pylori*^WT^ remains contradictory to the cholesterol accumulation under *H. pylori*^WT^ infection.

In order to deal with this contradiction, we explored to investigate the underlying mechanism at the protein level. Previous research showed that CagA is mainly present in the cytoskeleton and cell membrane^44^. We found that CagA could directly bind to mitochondrial protein CYP11A1, and they had a strong colocalization. This interaction hinted to us that a redistribution occurred in either CagA or CYP11A1 under *H. pylori*^WT^ infection. Further experiments confirmed the upregulation of CYP11A1 under the induction of CagA-positive *H. pylori* infection, with the majority of CYP11A1 being redistributed from the mitochondria to the extramitochondrial space.

Thus, the paradoxical phenomenon of simultaneous upregulation of cholesterol and cholesterol-catabolic CYP11A1 in the presence of *H. pylori*^WT^ infection was well explained. We concluded that CYP11A1 may be continuously transcriptionally upregulated due to a lack of mitochondrial CYP11A1 protein caused by its redistribution induced by CagA-CYP11A1 interaction under the infection of *H. pylori*^WT^. Further experiments revealed that *H. pylori* required CYP11A1 to induce mitochondrial cholesterol accumulation via CagA. Moreover, stronger colocalization of Mitotracker-Red and Filipin III was identified in *H. pylori*^WT^ infected group versus the uninfected group, illustrating the cholesterol induced by *H. pylori*^WT^ mainly accumulated in the mitochondria. To summarize, *H. pylori*^WT^ caused cholesterol accumulation in the mitochondria through CagA-mediated CYP11A1 redistribution outside the mitochondria.

On the other hand, accumulating cholesterol is closely associated with the development and progression of GC^62^-^66^. Recent studies have established low HDL-C and high LDL-C as independent risk factors of GC^35^, ^65^. Moreover, a meta-analysis of large observational studies also confirmed that a high-cholesterol diet increases the risk of GC^66^. Moreover, others also reported a rise in postoperative serum total cholesterol levels, which was linked to lower overall and recurrence-free survival rates in GC^63^. In addition, scientists have demonstrated the efficacy of statins (used for lower cholesterol) in reducing the likelihood of metachronous recurrence following endoscopic resection of early GC^67^. We also found that cholesterol stimuli could promote the malignant progression of GC *in vivo* and *in vitro* and protect GC cells against serum-induced apoptosis.

Because the cholesterol induced by *H. pylori*^WT^ was mainly present in the mitochondria, we explored the effect of mitochondrial cholesterol on the malignant progression of GC. The mitochondrial cholesterol upregulated by CYP11A1 knockdown could promote the proliferation of GC, while the statin stimuli could counteract this promotion. Conversely, the mitochondrial cholesterol depleted by CYP11A1 overexpression could inhibit the progression of GC, while cholesterol could abrogate this inhibition. These results also revealed that CYP11A1 impacted GC through varying mitochondrial cholesterol.

Mitochondrial cholesterol has been reported to inhibit mitophagy and apoptosis in other diseases, but no similar reports have been reported in GC cells^68^-^70^. We found that mitochondrial cholesterol accumulation caused by CYP11A1 knockdown could protect GC against starvation stimuli. Concretely, CYP11A1 knockdown-induced mitochondrial cholesterol accumulation could maintain mitochondrial homeostasis in starvation-treated cells, with a relatively more perfect status in mitochondrial size, function, and potential. Further experiments demonstrated that mitochondrial cholesterol inhibited mitophagy.

The relationship between *H. pylori* and the prognosis of GC has been debated in clinical research for a long time. Many scientists have proven through various clinical experiments that *H. pylori* positivity is associated with the survival and prognosis benefit of GC patients receiving S-1 adjuvant therapy^49^-^51^, ^71^-^74^. Furthermore, there are clinical studies that have reported conflicting research findings, specifically indicating that *H. pylori* infection does not have a favorable impact on the prognosis of patients with GC^45^, ^46^, ^48^, ^75^, ^76^. For example, a study about the impact of *H. pylori* eradication on the prognosis of patients with stage III/IV GC who underwent subtotal gastrectomy showed that *H. pylori* eradication failure was an independent risk factor for gastric cancer-related death (hazard ratio, HR = 3.41, *p* = 0.001) and recurrence (HR = 2.70, *p* = 0.005)^76^. These contradictory results cast doubt on the rationale of *H. pylori* eradication therapy in *H. pylori*-positive GC patients. However, previous basic research on *H. pylori* mainly focused on the pathogenesis of GC, which led to an unclear relationship between *H. pylori* and the progression/prognosis of GC. Our study successfully explains how *H. pylori* can promote the progression of GC through cholesterol metabolism, thereby providing a basis for anti-*H. pylori* therapy as an adjunct to GC treatment. More recently, a research team from Sun Yat-sen University Cancer Center released the results of a retrospective clinical study on adjuvant GC treatment with anti-*H. pylori* therapy, and the results also confirmed the benefits of anti-*H. pylori* therapy for GC patients^75^.

In conclusion, our study demonstrates that *H. pylori* can cause mitochondrial cholesterol accumulation through CYP11A1 redistribution outside the mitochondria, which is mediated by CagA-CYP11A1 interaction. Mitochondrial cholesterol can promote the malignant progression of GC and increase GC cells' resistance to harsh environments by inhibiting mitophagy and apoptosis. These findings suggest that patients with *H. pylori*^WT^-positive GC would benefit from eradicating *H. pylori* and taking medicine to lower cholesterol.

The limitation of this study is that it was not clear whether the redistribution of CYP11A1 occurred due to blocked input to the mitochondria after CYP11A1 protein synthesis or due to protein translocation from the mitochondria to the cytoplasm. In addition, the initial function enrichment of sequencing did not yield meaningful or interpretable results due to the relatively small number of genes selected for further investigation, as well as the inherent challenges in accurately capturing the complex and interconnected metabolic pathways using standard enrichment approaches. Therefore, we introduced two independent sequencing data from the GEO database and cholesterol metabolism-related gene sets to intersect with our sequencing data, ultimately yielding interpretable and meaningful targets. Although this screening process is common in scientific research, potential relevant targets or pathway may be overlooked.

## Materials and Methods

### Patients and tissue samples

Ten pairs of *H. pylori*-negative and -positive GC surgical specimens were selected for metabolomics and transcriptomics sequencing, and an additional 30 pairs of *H. pylori*-negative and -positive human GC tissues were chosen for qPCR detection of corresponding targets. All these patients had comparable baseline lipid profiles between *H. pylori*-negative and -positive subgroup. Moreover, all patients had not received *H. pylori* eradication treatment and those who had a history of metabolic diseases such as dyslipidemia were excluded. All included patients have histological evidence (Fish staining), 13C breath test, or gastroscopy report to specify the status of *H. pylori* infection.

Furthermore, data from 433 GC patients with known *H. pylori* status were obtained from the database to analyze the correlation between *H. pylori* and clinicopathological data. These GC patients were treated at the Department of Gastric Surgery, The First Affiliated Hospital of Nanjing Medical University, between 2018 and 2022, and the Department of Pathology determined the pathological types of the corresponding tissues. The study received approval from the Ethics Committee of the First Affiliated Hospital of Nanjing Medical University.

### *H. pylori* strains and culture

The study utilized *Helicobacter pylori* strain NCTC 11637 (GenBank accession number: AF202973; CagA positive strain: *H. pylori*^WT^) and its isogenic mutant CagA NCTC11637 (CagA negative strain: *H. pylori*^ΔCagA^) 77-80. These strains were cultivated on a brain-heart infusion medium supplemented with 10% rabbit blood under microaerophilic conditions (5% O2, 10% CO2, and 85% N2) at 37°C. AGS cells, transfected with plasmids and siRNAs, were then subjected to infection with *H. pylori*^WT^ and *H. pylori*^ΔCagA^, respectively, at a multiplicity of infection at 50 for 6 hours. Cells without infection were used as controls.

### Cell culture

The AGS and HGC-27 GC cell lines were procured from the Shanghai Institutes for Biological Sciences. These cell lines were cultured in RPMI 1640 medium and F12K medium, supplemented with 10% fetal bovine serum (Invitrogen, USA) and 1% penicillin/streptomycin (Gibco, USA). The cell cultures were maintained under standard conditions, with a CO2 concentration of 5% and a temperature of 37°C.

### Transfection and plasmid construction

GC cells were seeded in 6-well plates at the specified concentrations and allowed to incubate overnight. Transfection of plasmids and siRNA was accomplished using Lipofectamine 3000 (Thermo Fisher, USA). The supplementary materials provide the plasmid sequences, and the target sequences can be found in **Supplementary Table 2**. The efficiency knockdown of si-CYP11A1 or si-CYP19A1 was assessed through Western blot analysis (**Figure S7A and S7B**) and quantitative real-time polymerase chain reaction (qRT-PCR) (**Figure S7C and S7D**).

### RNA extraction and qRT-PCR

RNA extraction and quantitative real-time were performed as reported previously^72^. The sequences of primers are displayed in **Supplementary Table 3**.

### GC organoid model

GC tissues were dissected, minced, and digested with collagenase A after surgical procedures. The resulting cells were suspended in Matrigel (R&D Systems, Minneapolis, MN, USA) supplemented with growth factors. In a 24-well plate, the cell-Matrigel mixture was seeded and cultured in Organoid Growth Medium (StemCell Technologies, Canada). The growth and development of organoids were observed and recorded daily under a microscope.

### Western blot (WB)

Cells and tissues were lysed using RIPA Lysis Buffer (from Beyotime, Shanghai, China) following the provided guidelines. Then we transferred the lysates onto PVDF membranes (Millipore, Massachusetts, USA) after SDS-PAGE. These membranes were left overnight at 4°C with primary antibodies. Next, we treated the membranes with Super ECL Plus Kit (US EVERBRIGHT INC, Suzhou, China) after 2 h of incubation with corresponding secondary antibody. An antibody against a reference gene was used for normalization. **Supplementary Table 4** shows the primary antibodies and its corresponding observed molecular weight used in this study.

### Co-immunoprecipitation assays (Co-ip)

Treated cells were lysed using NP-40 Lysis Buffer (Beyotime) for 30 minutes. Subsequently, the lysates were centrifuged (15 minutes, 4°C, 12,000 × g) and incubated overnight at 4°C under rotational conditions with the specifically targeted antibodies: mouse anti-CagA (sc-28368 AF647, Santa Cruz Biotechnology) and rabbit anti-CYP11A1 (ab272494, Abcam).

Protein A/G PLUS-agarose (Santa Cruz and Biotechnology) was introduced to the samples and underwent overnight rotation at 4°C. Subsequently, the protein A/G PLUS-agarose was subjected to three consecutive washes, each with 1 ml of wash buffer (0.08% NP-40, 150 mM NaCl, 50 mM Tris-HCl (pH 8.0), and 5 mM MgCl2) for 20 minutes each at 4°C. The protein A/G PLUS-agarose was incubated at 95°C for nine minutes once the wash buffer was removed. Finally, the mixtures underwent centrifugation (15 minutes, 4°C, 12,000 × g), and the resulting supernatants were subjected to examination.

### Glutathione-S-transferase pull down (GST-pull down)

Briefly, the GST-tagged CYP11A1 recombinant protein (Abcam, ab132669) and the GST recombinant protein (Prospec, ENZ-393) were affixed to glutathione-Sepharose 4B beads (GE Healthcare, Little Chalfont, UK). Then, these bead-bound proteins were subsequently subjected to a 2-hour incubation at 4°C with the His-tagged CagA recombinant protein (Abcam, ab224836). After that, the complexes were washed four times with GST-binding buffer and eluted five times with TNGT elution buffer (0.5 ml). The elution fractions were analyzed with the indicated antibodies by WB.

### Cell and tissue Immunofluorescence

For cell immunofluorescence, treated cells were seeded on confocal dishes at a concentration of 3 × 10^4^ cells per milliliter. Subsequently, the GC cells were fixed (4% paraformaldehyde) for 5 minutes, permeated (0.1% Triton X-100) for 20 minutes, and blocked (5% bovine serum albumin (BSA)) for 1 hour. The cells were then subjected to overnight incubation with the corresponding primary antibodies. Following this, the cells were exposed to corresponding secondary antibodies for 1 hour. Finally, the GC cells were counterstained with 4',6-diamidino-2-phenylindole (DAPI) (KGA215-10, KeyGEN BioTECH) for 10 minutes and photographed using a fluorescence microscope. All antibodies were diluted according to the manufacturer's instructions.

For tissue Immunofluorescence, embed fresh tumor tissues in OTC (Cat:4583, SAKURA Tissue-Tek O.C.T. Compound) medium were frozen and serially sectioned with 10 μm thin (LEICACM1950). These sections were then fixed using 4% paraformaldehyde for 1 h. Fixed sections were subject to 3% H_2_O_2_ for 30 min at room temperature and 2% BSA (9048-46-8, Sigma-Aldrich). Next, they are incubated with primary antibodies overnight at 4°C. The sections were mounted and photographed (Thunder Imager Fast High-Resolution Inverted Fluorescence Imaging System, THUNDER DMi8, LEICA) after incubating the corresponding secondary antibodies and dapi (KGA215-10, KeyGEN BioTECH). Specific information of antibodies is provided in **Supplementary Table 3**.

### Filipin III cholesterol staining

Filipin III cholesterol staining was performed using frozen section total cholesterol (esterase method) Filipino (FILIPIN) fluorescence staining kit (GMS80079.3, GENMED) and Cellular-total cholesterol (esterase method) Filipino (FILIPIN) fluorescence staining kit (GMS80079.1, GENMED) according to the manufacturer's instructions.

### Transmission electron microscopy

The treated were digested (trypsin) and separated by centrifugation at 300 g for 5 minutes. Then, the cells were mixed with serum in a microcentrifuge tube. Next, the cells were preserved in EM fixation buffer overnight at 4°C. Following fixation, the samples were stained using OsO4 (1%) and subsequently cut into extremely thin sections. These sections were finally examined using electron microscopy (JEM-F200, Japan).

### Colony formation assays

A six-well plate with 2 mL complete media contained 1000 cells per well. With 4% paraformaldehyde, visible colonies were fixed. The plate was then gently flushed with tap water to remove the fixation liquid. Subsequently, the colonies were subjected to staining with crystal violet, and photographic documentation took place precisely 14 days after desiccation at ambient room temperature.

### CCK8

GC cells treated with the specified treatment were seeded in 96-well plates at a density of 5 × 10^3^ cells per well and incubated for 4 hours. The Cell Counting Kit-8 (HY-K0301, MCE, China) was employed to evaluate cell proliferation capability. The absorbance was recorded at 450 nm each day over a continuous span of 5 days, at consistent time intervals.

### 5-Ethynyl-2′-deoxyuridine assays (EdU assays)

The Click-iT Cell-Light EdU Apollo567 *In vitro* Kit (Ribobio, Guangzhou, China) was employed to perform the EdU assay following the manufacturer's protocols. Briefly, digested cells were plated in a 96-well plate at a density of 5000 cells per well. EdU was then introduced into the medium at a final concentration of 50 mol/L and incubated with the cells for 3 hours. Subsequently, the cells were fixed using 4% paraformaldehyde in PBS, and the reaction was halted with 50 μL of glycine solution (2 mg/mL). Lastly, the cells were stained with Hoechst 33342 and examined using a fluorescence microscope (Thunder Imager Fast High-Resolution Inverted Fluorescence Imaging System, THUNDER DMi8, LEICA).

### Apoptosis experiment

After treatment, GC cells were digested using Trypsin (25200072, GIBCO), and the whole-cell lysates were collected. Subsequently, the lysates were incubated with propidium iodide (PI) and annexin V for 20 minutes. Finally, the cells were analyzed using the CELL Quest software (BD Biosciences, USA), and the proportion of apoptosis was represented by the corresponding quadrant on the graph.

### Luciferase reporter assay

The dual-luciferase reporter assay was generated using the Dual-Luciferase Reporter Assay System (Promega) based on the manufacturer's protocol. Briefly, GC cells were seeded in the 24-well plates and transfected with the luciferase vector fused to the CYP11A1. Relative luciferase activity was normalized to Renilla luciferase activity.

### Immunohistochemistry

Human GC tissue from surgical specimens and nude subcutaneous tumors tissues were fixed and embedded in paraffin for immunohistochemistry (IHC). The sections (4 μm) derived from paraffin-embedded tissues were incubated with the indicated antibody overnight at 4°C and then with a secondary antibody HRP conjugate at room temperature for 1 h.

### Animal studies

A total of 6 × 10^6^ logarithmically growing HGC-27 cells pretreated were resuspended in 100 μL PBS and injected subcutaneously into the flank of 6-week-old male nude mice. The commencement of treatments was scheduled when the tumor volume reached approximately 300 mm^3^. (1) The mice were randomly divided into two groups: a normal diet (ND)group and a high cholesterol diet (HCD) group (from Beijing Ke'ao Xieli Feed Co., Ltd) (n=5 mice/group). (2) Pretreated HGC-27 cells with CYP11A1 plasmids or control were used to construct subcutaneous tumors in mice (n= 5 mice/group). For CYP11A1 overexpression group, mice were chowed with high cholesterol diet. (3) HGC-27 cells transfected with si-CYP11A1 or si-NC were used to construct subcutaneous tumors in nude mice (n= 5 mice/group). For CYP11A1 knock-down group, mice were treated with atorvastatin (5mg/kg/d) for 2 weeks (n=5). (4) GC cells co-cultured with different *H. pylori* strains were used to construct subcutaneous tumors (n= 5 mice/group). The entire experimental protocol was conducted in accordance with the guidelines of the local institutional animal care and use committee (Ethics number: IACUC-2207029).

## Supplementary Material

Supplementary figures and tables.

## Abbreviations

CagA: Cytotoxin-associated gene A
DEGs: differentially expressed genes
ECM: extracellular matrix
GC: gastric cancer
GEO: Gene Expression Omnibus database
*H. pylori*: *Helicobacter pylori*
*H. pylori* ^△CagA^: *Helicobacter pylori* strain with CagA deficiency
*H. pylori* ^WT^: *Helicobacter pylori* strain without CagA deficiency
HCD: high cholesterol diet
HDL: high-density lipoprotein
IF: Immunofluorescence
IHC: Immunohistochemistry
KEGG: Kyoto Encyclopedia of Genes and Genomes
LDL: low-density lipoprotein
MSigDB: Molecular Signature Database
ND: normal diet
qRT-PCR: Quantitative real-time polymerase chain reaction
T4SS: the type IV secretion system
TC: total cholesterol
TME: tumor microenvironment
WB: western blot
WHO: World Health Organization
FISH: Fluorescence *in situ* hybridization staining
