# Supplementary material for: Helicobacter pylori promotes gastric cancer through CagA-mediated mitochondrial cholesterol accumulation by targeting CYP11A1 redistribution
Source: Int J Biol Sci. 2024 Jul 15;20(10):4007–28. doi: 10.7150/ijbs.96425 (PMC11302876; doi:10.7150/ijbs.96425)
Supplement: Supplementary file 1 — Supplementary figures and tables. [file ijbsv20p4007s1.pdf]

Supplementary table 1. Relationships between H.pylori infection and clinicopathological characteristics in 433 GC patients.

Parameter	Cases	*H. pylori* infection		*p* value
		Negative	Positive	
**Age**				0.8289
<60	343	114	229	
≥60	90	31	59	
**Gender**				0.0669
Male	215	63	152	
Female	218	82	136	
**Differentiation degree**				0.6369
Poorly	229	79	150	
Highly and moderately	204	66	138	
**Invasive depth**				0.2565
T2	14	3	11	
T3	196	60	136	
T4	223	82	141	
**TNM stage**				0.8397
I/II	221	75	146	
III/IV	212	70	142	
**Preoperative Total cholesterol**				0.6008
<6	416	138	278	
≥6	17	7	10	
**Preoperative HDL-C**				0.3017
<1.0	144	53	91	
≥1.0	289	92	197	
**Preoperative LDL-C**				0.0430
<4	403	140	263	
≥4	30	5	25	
**Postoperative Total cholesterol**				0.0414
<6	419	144	275	
≥6	14	1	13	
**Postoperative HDL-C**				<0.0001
<1.0	205	41	164	
≥1.0	228	104	124	
**Postoperative LDL-C**				0.0040
<4	408	143	265	
≥4	25	2	23	

Supplementary table 2. The siRNA sequences in this study.

name	sequences
Si-CYP11A1-1	ACTCGACCCTTCTTTATGA
Si-CYP11A1-2	GAAAGCCATCCTCGTTACA
Si-CYP11A1-3	AGTGCATCGGTATGCATGA
Si-CYP19A1-1	GCTGCATGGGACGTGATTT
Si-CYP19A1-2	TGGTTCTTCGAGATTACAT
Si-CYP19A1-3	ACATGGCCACGATGCTACA

Supplementary table 3. The primers in this study.

Primer name	Sequence(5’-3’)
CYP11a1-F	GGATGCTGGAGGAAGTAGTGAAC
CYP11a1-R	TGGTGAACACTTCCTTTCTGTCT
CYP19a1-F	GCAAAGCACCCTAATGTTGAAGA
CYP19a1-R	CGAGTCTGTGCATCCTTCCAATA
WNT10a-F	ATCCACGAATGCCAACACCAATT
WNT10a-R	CTCTCGGAAACCTCTGCTGAAGA
GATA3-F	CTCCTCCTCCTCTCTGCTCTTC
GATA3-R	AAGCAAAGGTGAGCAAAGGAGAA
EGFR-F	CTGGGTGCGGAAGAGAAAGAATA
EGFR-R	CCAAAGGTCATCAACTCCCAAAC
GAPDH-F	GGAGCGAGATCCCTCCAAAAT
GAPDH-R	GGCTGTTGTCATACTTCTCATGG

Supplementary table 4. The antibodies in this study.

Name	Manufacturer	Catalog	observed molecular weight
anti-CYP11A1	abcam	ab272494	50 kDa
anti-CYP11A1	Proteintech	13363-1-AP	50 kDa
anti-CYP19A1	Affinity	BF8059	43-50 kDa
anti-cagA	Santa Cruz	sc-28368	120 kDa
anti-cagA	Santa Cruz	sc-28368 AF647	120 kDa
anti-GAPDH	Proteintech	60004-1-Ig	36 kDa
anti-GST	Santa Cruz	sc-138	26 kDa
anti-LC3	CST	4108S	14/16 kDa
anti-p62	CST	23214S	62 kDa
anti-PINK1	Proteintech	23274-1-AP	65 kDa
anti-Parkin	CST	#32833	50 kDa
anti-Tom20	Abcam	ab186735	16 kDa
Ani-Ki67	CST	34330SF	
VeriBlot for IP Detection reagents (HRP)	Abcam	ab131366	
GAPDH Rabbit pAb	ABclonal	AC001	36 kDa
Mouse IgG (H+L) Highly Cross-Adsorbed Secondary Antibody	Invitrogen	A32723	
Donkey anti-Rabbit IgG (H+L) Highly Cross-Adsorbed Secondary Antibody	Invitrogen	A-11034	

Supplementary Figures and legends

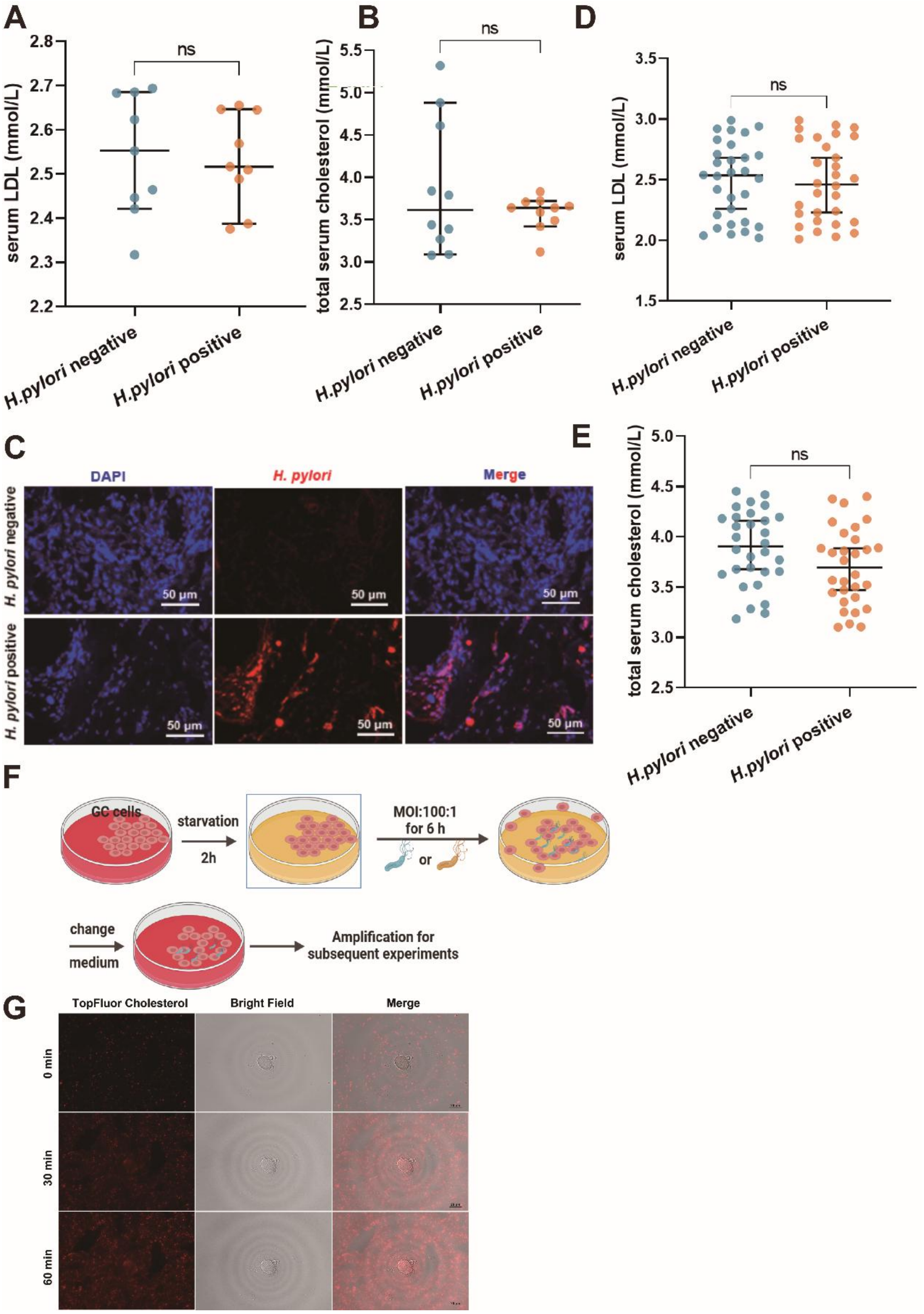

Fig. S1. The baseline serum lipid profiles of patients and the co-culture of GC cells with *H. pylori* strains.

A-B The scatter plots showed the distributions of the high-density lipoprotein cholesterol (LDL) (A) and the total serum cholesterol (B) from GC patients whose surgical specimens

were selected for sequencing. **C** The typical images of GC tissues under different *H. pylori* infection status as determined by FISH fluorescence staining with *H. pylori* probes. **D-E** The scatter plots showed the distributions of the high-density lipoprotein cholesterol (LDL) (A) and the total serum cholesterol (B) from GC patients whose surgical specimens were use to examine the cholesterol content. **F** Schematic diagram of co-culture of Helicobacter pylori and GC cells. Initially, GC cells in the logarithmic growth phase were subjected to serum deprivation by incubating them in a serum-free medium for a duration of 2 hours following fluid exchange. Subsequently, the H. pylori strain was introduced to the starved cells at an infection ratio of 100:1 (bacteria: cells). Following a 6-hour co-culture treatment, the medium was once again replaced, and the cells were further expanded to a specific cell density to facilitate subsequent experimental procedures. **G** Visualization of fluorescent cholesterol uptake in HGC-27 cells using live cell imaging. Data and error bars were shown as mean ± SD of triplicate independent replicate experiments. For the assessment of data passing independence, normality, and homogeneity of variance, the Student's t-test was employed to compare the differences between the two sets of data. Nonparametric tests were utilized in cases where the aforementioned conditions were not met. Significant flags and *p*-values are intricately linked in the following manner: (*$p < 0.05$; **$p < 0.01$; ***$p < 0.001$; ****$p < 0.001$).

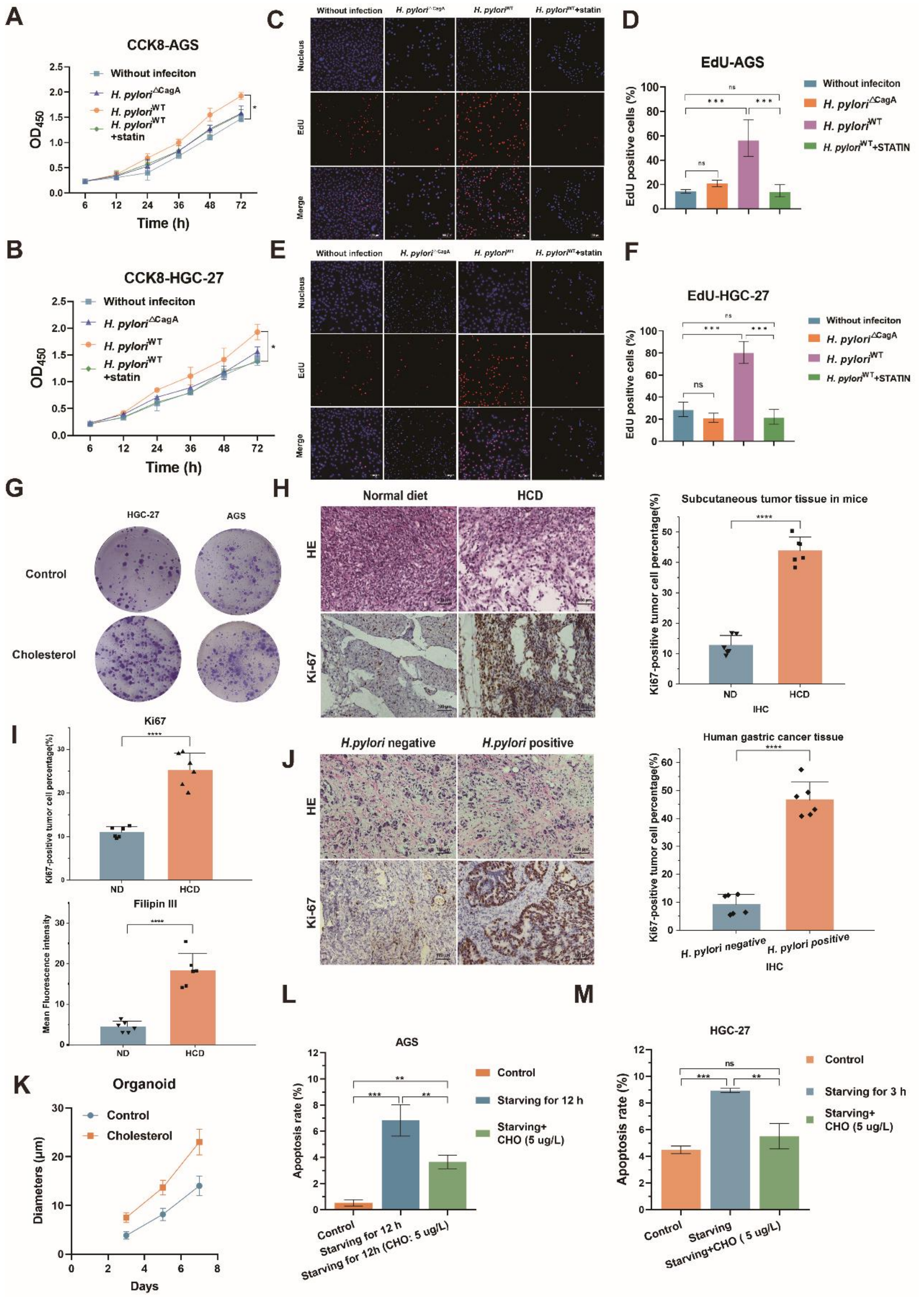

Fig.S2. The effect of cholesterol on the proliferation and apoptosis of GC

A-B CCK8 assays were performed in AGS cells and HGC-27 cells in a combination treatments of different *H. pylori* infeciton and AVT stimuli. **C-F** EdU experiments were conducted in the same groups as in A and B. **G** The colony formation assays of GC cells alone or with the stimuli of cholesterol (5 ug/L). **H** The HE and Ki-67 immunohistochemical staining of subcutaneous tumor constructed from HGC-27 cells in mice chowed with

normal diet (ND) or high-cholesterol diet (HCD) (left) and the quantification of Ki67-positive tumor cells (right). **I** The quantification of Ki67-positive tumor cells (upper)and Filipin **III** staining (lower) in **Figure S2E.** **J** The HE and Ki-67 immunohistochemical staining of human GC tissues with or without *H. pylori* infection (left) and the quantification of Ki67-positive tumor cell percentage (right). **K** Diameter of organoids constructed from the surgical specimen of human GC. **L-M** The quantification of the effect of cholesterol on the apoptosis rate of AGS (K) and HGC-27 (L) cells treated by serum-free medium by flow cytometry assay.

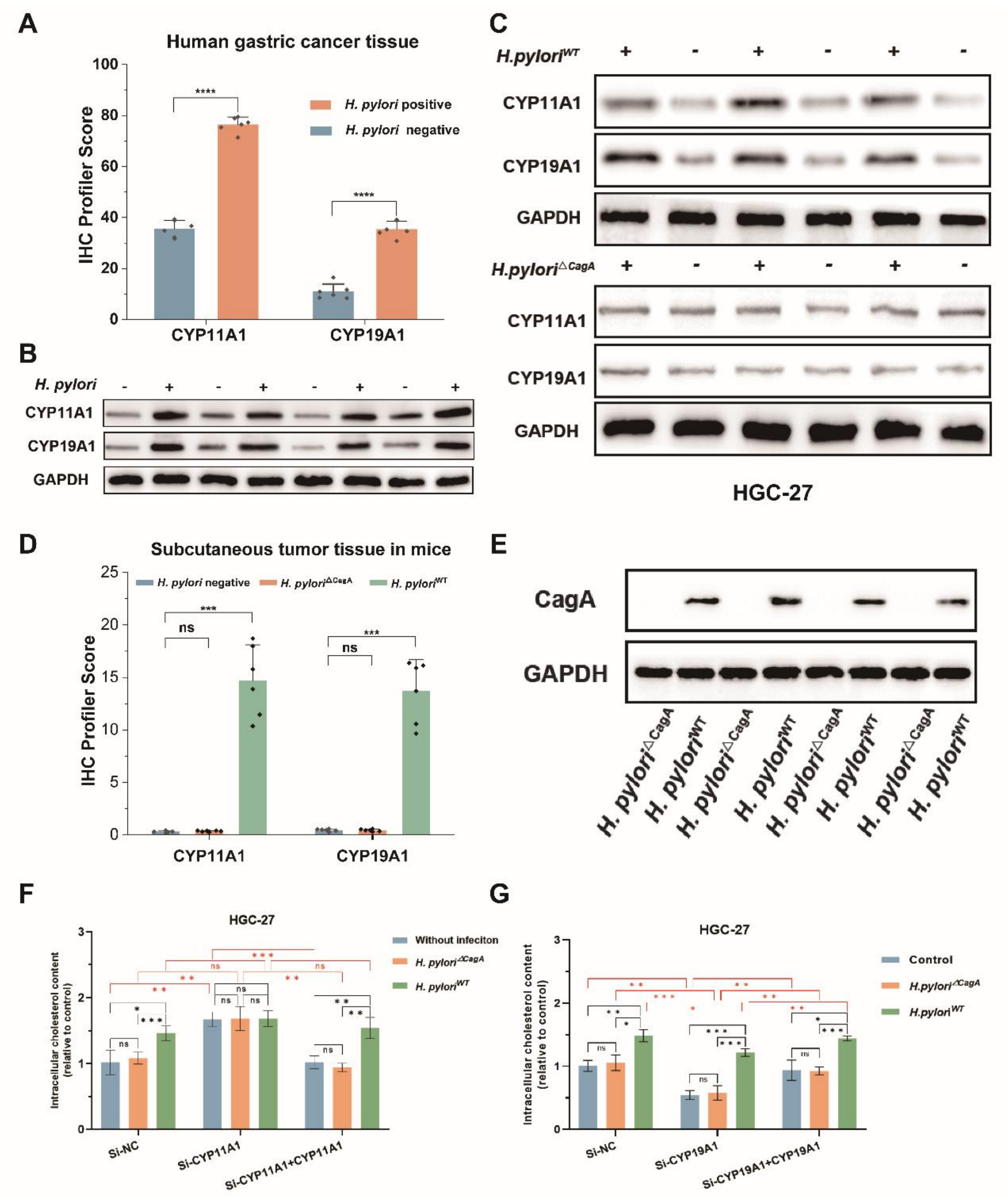

Fig.S3. *H. pylori* induced the expression of CYP11A1/CYP19A1 and caused cholesterol accumulation in a CagA-dependent manner.

A The quantification of **Figure 3G**. **B** The examination of CYP11A1 and CYP19A1 by WB in human GC. **C** The examination of CYP11A1 and CYP19A1 by WB in HGC-27 cells co-cultured with different *H. pylori* strains. **D** The quantification of **Figure 3I. E** The examination of CagA by WB in HGC-27 cells co-cultured with *H. pylori*$^{△CagA}$ and *H. pylori*^{WT}. **F-G** The relative cholesterol content was varying in HGC-27 cells with the manipulation of CYP11A1 (C) or CYP19A1 (D) under the infection of different *H. pylori* strains. Data and error bars were shown as mean ± SD of triplicate independent replicate experiments. For the assessment of data passing independence, normality, and homogeneity of variance, a mixed-design analysis of variance was used for pairwise comparisons. Nonparametric tests were utilized in cases where the aforementioned conditions were not

met. Significant flags and p-values are intricately linked in the following manner: (*$p < 0.05$; **$p < 0.01$; ***$p < 0.001$; ****$p < 0.001$).

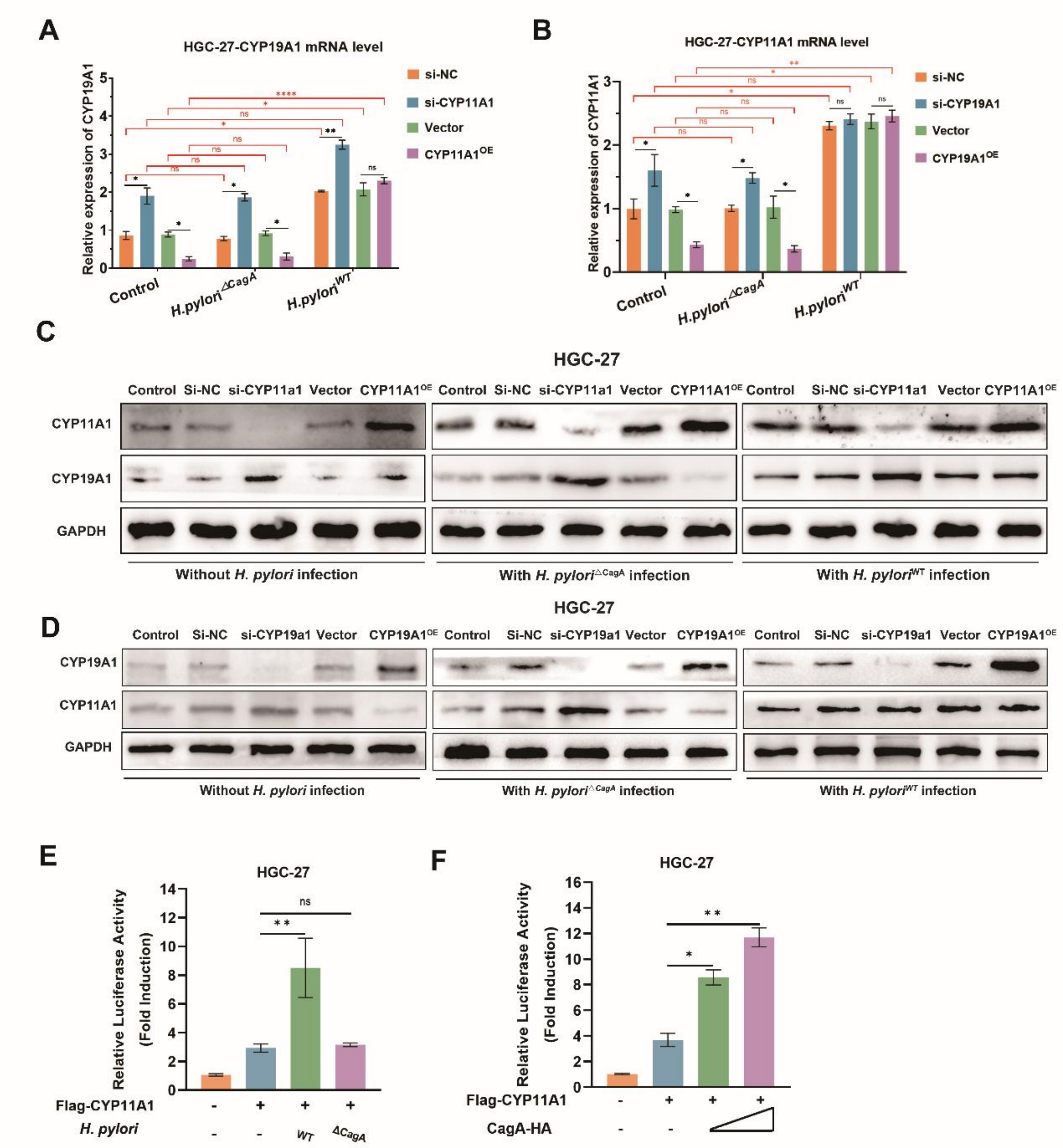

Fig.S4. The *H. pylori*^{WT} disrupted the negative regulatory relationships between CYP11A1 and CYP19A1 in a CagA-dependent manner.

A The effects of different CYP11A1 levels on CYP19A1 were examined by PCR in HGC-27 cells alone or those pretreated by various *H. pylori* strains infection. **B** The influence of varying CYP19A1 levels on CYP11A1 were demonstrated by PCR in HGC-27 cells alone or those infected with different *H. pylori* strains. **C** The effects of CYP11A1 on CYP19A1 were examined by WB in HGC-27 cells alone and those co-cultured with different *H. pylori* strains. **D** The effects of CYP19A1 on CYP11A1 were examined by WB in HGC-27 cells alone or those infected with different *H. pylori* strains. **E** Luciferase reporter assay were conducted in transfected HGC-27 cells under the infection of different *H. pylori* strains. **F** Luciferase reporter assay were performed in HGC-27 cells transfected with indicated plasmids for 48 h. Data and error bars were shown as mean ± SD of triplicate independent replicate experiments. For the assessment of data passing independence, normality, and homogeneity of variance, the Student's t-test was employed

to compare the differences between the two sets of data. A mixed-design analysis of variance or one-way analysis of variance was used for pairwise comparisons. Nonparametric tests were utilized in cases where the aforementioned conditions were not met. Significant flags and p-values are intricately linked in the following manner: (*p < 0.05; **p < 0.01; ***p < 0.001; ****p < 0.001).

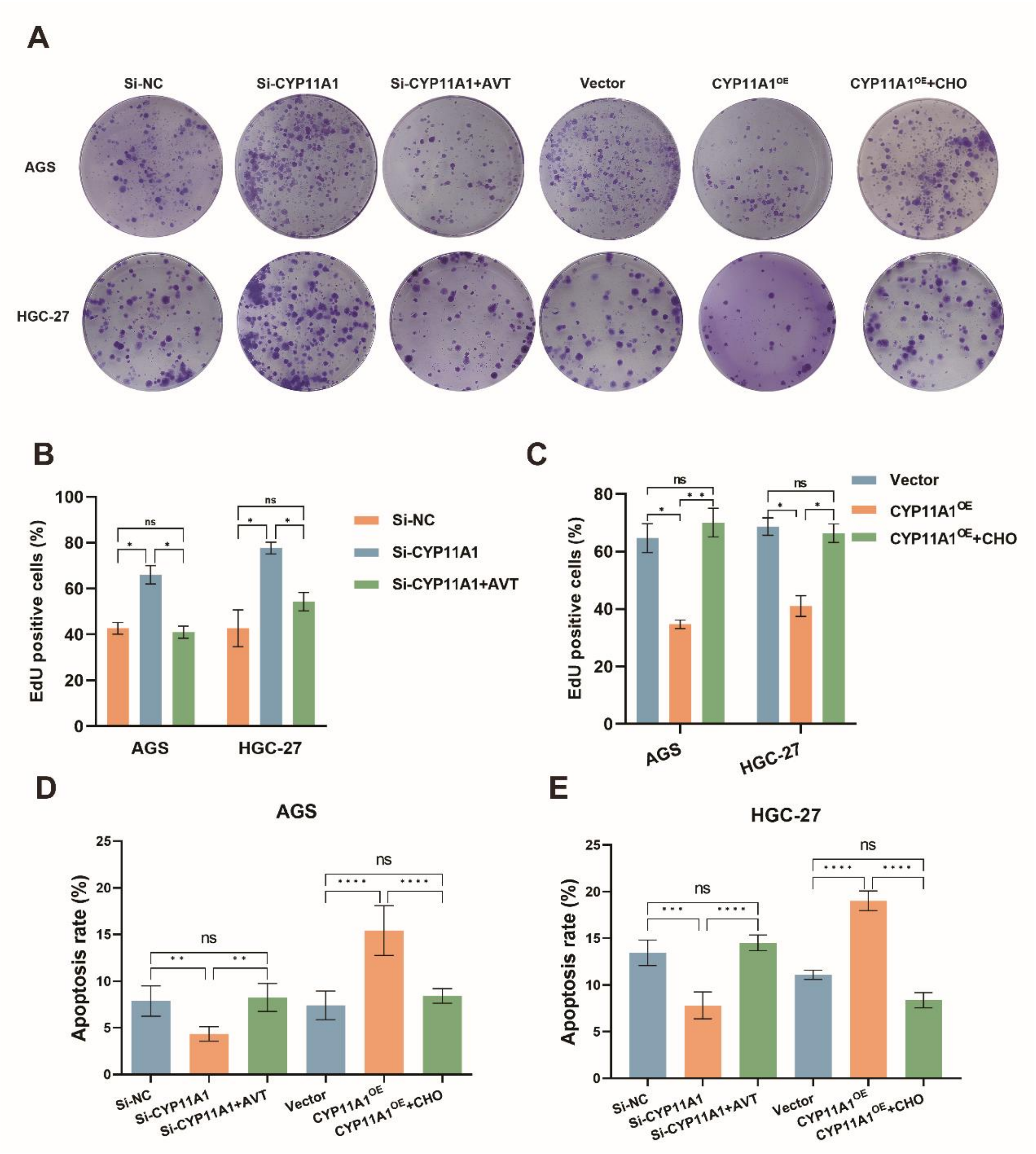

Fig.S5. CYP11A1 impacted the proliferation of GC cells and the serum-free medium-induced apoptosis through the regulation of cholesterol.

A The colony formation assays were used to show the impact of CYP11A1 on the proliferation of GC cells. **B-C** The quantification of **Figure 5G** and **5H**. **D-E** The quantification of Figure 5J and 5K. Data and error bars were shown as mean ± SD of triplicate independent replicate experiments. For the assessment of data passing independence, normality, and homogeneity of variance, the Student's t-test was employed to compare the differences between the two sets of data. One-way analysis of variance was used for pairwise comparisons. Nonparametric tests were utilized in cases where the aforementioned conditions were not met. Significant flags and p-values are intricately linked in the following manner: (*p < 0.05; **p < 0.01; ***p < 0.001; ****p < 0.001).

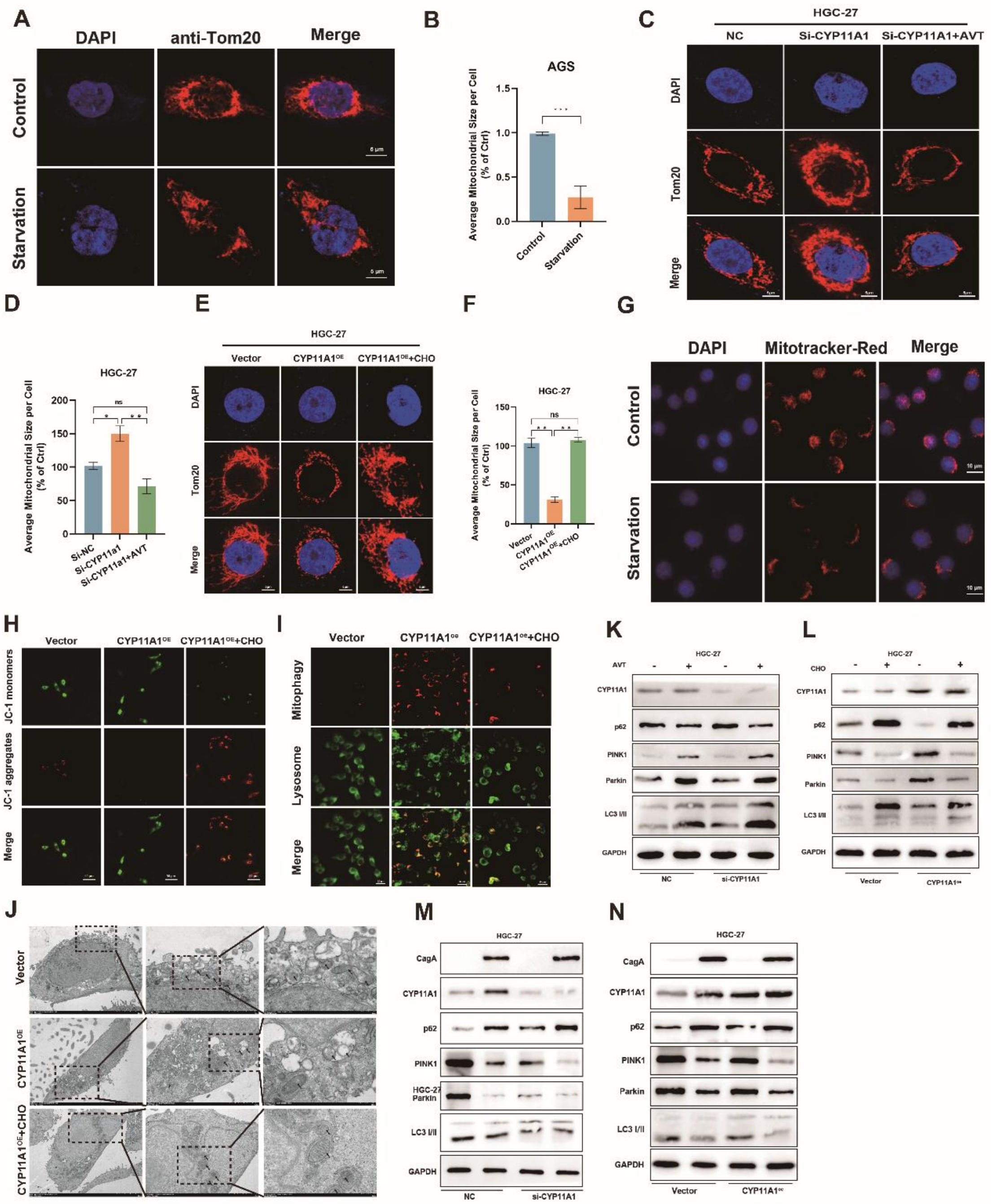

Fig.S6. CYP11A1 suppress mitophagy via decreasing mitochondrial cholesterol content.

AGS cells and HGC-27 cells were transfected by CYP11A1 or si-CYP11A1 or the corresponding control. Subsequently, they were treated by serum-free medium for the indicated time (AGS cells: 12 h； HGC-27 cells: 3 h). Cholesterol stimuli (5 ug/L) was simultaneously administered in CYP11A1-overexpressed GC cells. AVT (5 uM, 24h)was used to treat CYP11A1-knockdown GC cells. **A-B** The immunofluorescence staining of anti-Tom20 represented the total mitochondrial size in AGS cells alone or in those pretreated with serum-free medium for 12 h (A) and the quantification of A (B). **C-F** The immunofluorescence staining of anti-Tom20 represented the total mitochondrial size in HGC-27 cells with CYP11A1 knockdown (C) or overexpression (E) and the average

mitochondrial sizes of C and E were quantified (D, F, respectively). **G** The Mitotracker-Red staining represented the functional mitochondria in AGS cells or in those pretreated with serum-free medium for 12 h. **H** The immunofluorescence staining using JC-1 represented the mitochondrial potential in treated AGS cells with CYP11A1 overexpression and/or cholesterol stimuli. **I** Co-staining of mitophagy dye and lysosomal dye was used to detect the mitophagy. **J** Electron microscopy showed representative mitophagy induced by starvation in cells with CYP11A1 overexpression and/or cholesterol stimuli. **K-L** Examination of mitophagy markers by WB in knockdown group alone or with AVT stimuli (K) and in overexpression group alone or with cholesterol stimuli (L). **M-N** Examination of mitophagy markers by WB in infected HGC-27 cells with CYP11A1 knockdown (M) or overexpression (N) under different *H. pylori*^{WT} infeciton status.

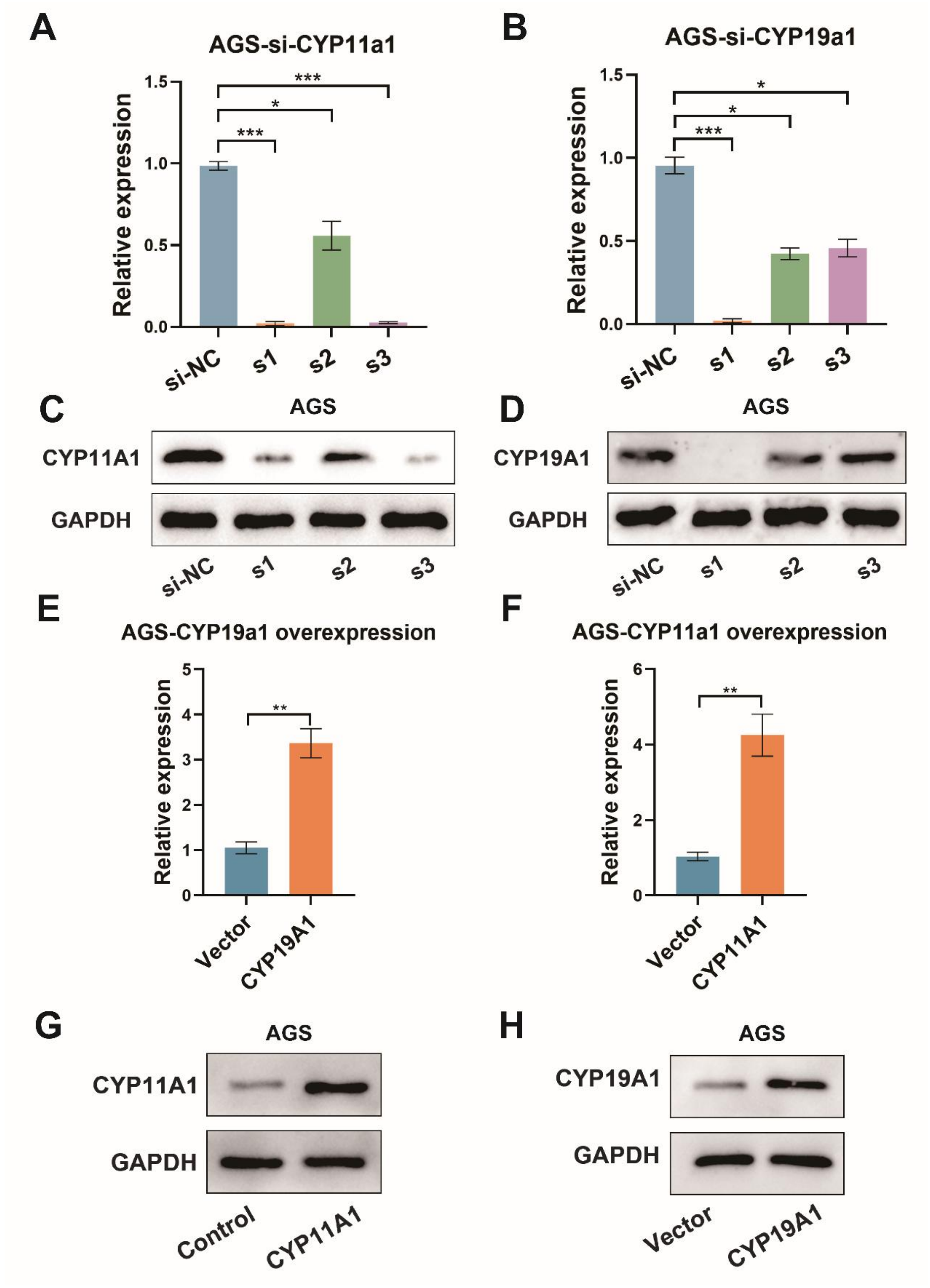

Fig.S7. The verification of CYP11A1 or CYP19A1 knockdown and overexpression by PCR and WB.
